# Optimizing ionic transport in argyrodites: a unified view on the role of sulfur/halide distribution and local environments†

**DOI:** 10.1039/d4ta04628e

**Published:** 2024-09-09

**Authors:** Anastasia K. Lavrinenko, Theodosios Famprikis, James A. Quirk, Victor Landgraf, Pedro B. Groszewicz, Jouke R. Heringa, Stef Smeets, Victor Azizi, Simone Ciarella, James A. Dawson, Marnix Wagemaker, Alexandros Vasileiadis

**Affiliations:** a Storage of Electrochemical Energy, Department of Radiation Science and Technology, Faculty of Applied Sciences, Delft University of Technology Mekelweg 15 2629JB Delft The Netherlands m.wagemaker@tudelft.nl a.vasileiadis@tudelft.nl; b Chemistry – School of Natural and Environmental Sciences, Newcastle University Newcastle upon Tyne NE1 7RU UK; c Helmholtz Zentrum Berlin für Materialien und Energie Hahn-Meitner-Platz 1 14109 Berlin Germany; d Netherlands eScience Center Science Park 402 1098 XH Amsterdam The Netherlands

## Abstract

Understanding diffusion mechanisms in solid electrolytes is crucial for advancing solid-state battery technologies. This study investigates the role of structural disorder in Li_7−*x*_PS_6−*x*_Br_*x*_ argyrodites using *ab initio* molecular dynamics, focusing on the correlation between key structural descriptors and Li-ion conductivity. Commonly suggested parameters, such as configurational entropy, bromide site occupancy, and bromine content, correlate with Li-ion diffusivity but do not consistently explain conductivity trends. We find that a uniform distribution of bromine and sulfur ions across the 4a and 4d sublattices is critical for achieving high conductivity by facilitating optimal lithium jump activation energies, anion-lithium distances, and charge distribution. Additionally, we introduce the ionic potential as a simple descriptor that predicts argyrodite conductivity by assessing the interaction strength between cations and anions. By analyzing the correlation between ionic potential and conductivity for a range of argyrodite compositions published over the past decade, we demonstrate its broad applicability. Minimizing and equalizing ionic potentials across both sublattices enhances conductivity by reducing the strength of anion-lithium interactions. Our analysis of local environments coordinating Li jumps reveals that balancing high and low-energy pathways is crucial for enabling macroscopic diffusion, supported by investigating percolating pathways. This study highlights the significance of the anionic framework in lithium mobility and informs the design of solid electrolytes for improved energy storage systems.

## Abstract 2

Uniform bromine and sulfur ion distribution across sublattices in Li_7−*x*_PS_6−*x*_Br_*x*_ argyrodites is key to high Li-ion conductivity. It enhances Li-ion transport by balancing high- and low-energy jump environments, enabling lower energy percolation.

## Graphical abstract

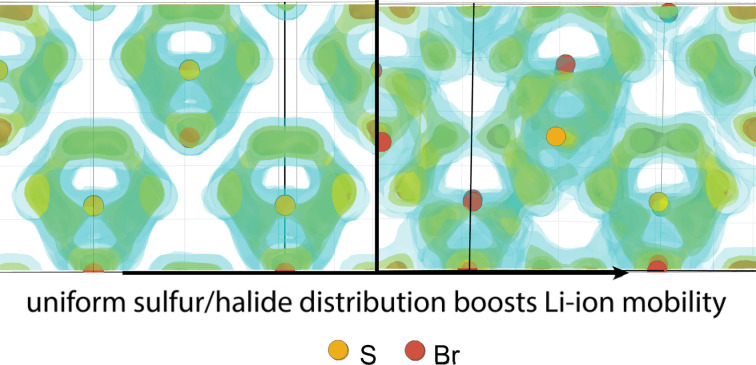

## Introduction

Solid-state batteries (SSBs) are attracting considerable attention as a potential energy storage technology, offering significant benefits over conventional liquid-electrolyte-based counterparts. SSBs show promise for future use in electric vehicles and portable electronics due to their increased safety, high energy density, and broader range of operating conditions.^1–6^ However, achieving the necessary high ionic conductivity for efficient energy transfer at ambient temperatures remains a significant challenge in solid electrolyte materials. Only a few electrolyte families qualify as superionic conductors, including NASICON-type, LISICON-type, garnets, argyrodites, perovskites, lithium nitrides, and halides.^1,7–11^ Among them, argyrodite-structured sulfide solid electrolytes stand out due to their exceptional conductivity—of the order of 10 mS cm^−1^, which rivals that of conventional liquid electrolytes—and their mechanical softness, contributing to reduced interfacial resistance and simplifying the manufacturing process.^2,9,12^ The ongoing quest in this field involves improving the performance of existing materials and discovering new electrolytes that enable fast ionic transport while maintaining favorable electrochemical stability and processability. Understanding the intricate mechanisms that drive high conductivity guides the design and optimization of solid electrolytes, paving the way for next-generation solid-state batteries.

Recent studies have proposed several strategies to improve the ionic conductivity of argyrodite-type materials. Pioneering research highlighted the strong connection between the diffusion of Li^+^ ions and mixing S^2−^ and X^−^ ions at ionic sublattices in argyrodite compounds Li_6_PS_5_X (where X can be Cl, Br, or I).^13–23^ Notably, S^2−^/X^−^ site mixing is observed in Li_6_PS_5_Cl and Li_6_PS_5_Br, reducing the activation energy and thereby enhancing lithium ion conductivity.^17,24^ Conversely, Li_6_PS_5_I does not exhibit S^2−^/X^−^ mixing when prepared with traditional synthesis techniques, due to a significant mismatch in ionic radii between S^2−^ and I^−^, resulting in lower ionic conductivity compared to its Cl and Br counterparts.^16,25,26^ Introduction of S^2−^/I^−^ disorder in the argyrodite structure improves conductivity in comparison to the ordered arrangement of sulfur and iodine anions.^15,16,22,25,27^

Another effective strategy for improving the conductivity is increasing the halide content through aliovalent substitutions. Molecular dynamics simulations and experimental investigations have demonstrated that introducing Li^+^ vacancies by substituting S^2−^ with halides significantly increases ionic conductivity. Specifically, altering the halide content in argyrodites (Li_6−*x*_PS_5−*x*_(Cl, Br, I)_1+*x*_) lowers the activation barrier, leading to a substantial increase in conductivity.^13,17–19,28^ Further, aliovalent substitutions, such as replacing P^5+^ with Ge^4+^, alter the lattice parameters and increase Li^+^ conductivity by enabling long-range diffusion.^11,15,29,30^ Studies on replacing P^5+^ with Si,^11,27,29,31^ Sb,^29^ and Sn^11,29^ have also shown significant improvements in conductivity, further indicating that the energy landscape of lithium argyrodites can be tailored to promote higher Li^+^ mobility through tailoring the local disorder and elemental substitutions. Isovalent substitutions of sulfur can achieve a similar effect. For example, replacing S^2−^ with larger, more polarizable ions like Se^2−^ can also enhance conductivity by influencing Se^2−^/X^−^ disorder and widening Li^+^ diffusion pathways.^21,32,33^

The origins of rapid ion conduction in argyrodite-type electrolytes are diverse and challenging to unravel, as evidenced by the ongoing debate over the mechanisms responsible for their enhanced diffusion properties. Following the discussion above, several factors have been highlighted. Li^+^ diffusivity correlates with S^2−^/X^−^ disorder and halide occupation on the anion sublattices, which are considered crucial factors in improving conductivity. Such site disorder impacts the average anionic charge distribution, affecting electrostatic interactions in the structure, thereby impacting lithium diffusion.^12,22,28,30,34–36^ In relation to this, recent studies have argued that ion conductivity increases with an increase in the configurational entropy at both cation and anion sublattice, proposing a direct link between high entropy and high diffusivity.^37–40^ Another aspect that has been brought forward is lattice softness, suggesting that a softer and more polarizable anion lattice enhances conductivity by affecting both the migration barrier for the diffusing cation and the Arrhenius prefactor, putting forward a nonlinear correlation with conductivity that demands further exploration.^10,24^

From the above, it is clear that a diversity in mechanisms and descriptors is considered and debated, aiming to understand the Li-ion mobility in argyrodite-type materials. The underlying question remains unanswered: is it a singular property or a collection of interrelated characteristics that govern the relationship between structure and Li-ion dynamics? This question motivates us to analyze the complex relationship between anion sublattice disorder and its impact on ionic conductivity. In addressing this challenge, our study employs density functional theory (DFT) and *ab initio* molecular dynamics (AIMD) to analyze the diffusion mechanisms at play. With Li_7−*x*_PS_6−*x*_Br_*x*_ serving as a model system, we meticulously explore the lithium-ion diffusion pathways, investigate the activation energy landscape shaped by the anion configuration, and examine how disorder modulates conductivity. We deconvolute the factors influencing ionic conductivity, explaining the roles of site disorder and local environment, ultimately introducing innovative descriptors designed to decode the experimental trends observed in argyrodite materials. This detailed comparison with reported argyrodite compositions, a review-like analysis between calculations and experiments, provides a unified, in-depth understanding of how specific atomic arrangements affect ion mobility, marking a significant step toward refining energy storage solutions by optimizing electrolyte design.

## Computational details

### Generation of structures

**Fig. 1:**
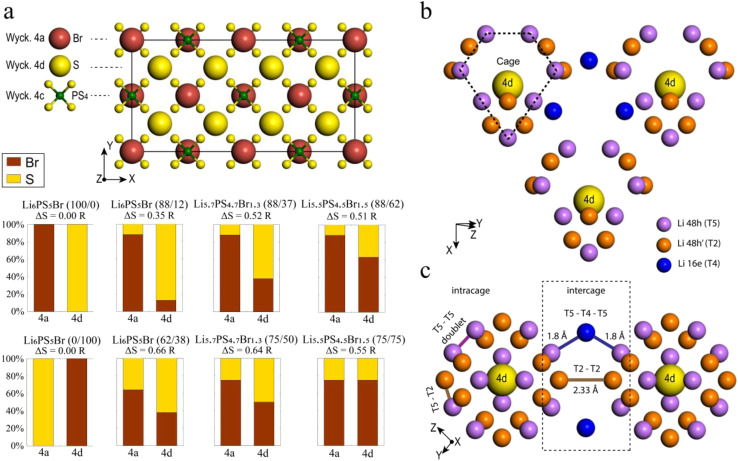
Structural details of argyrodite. (a) Anion framework in a 2 × 1 × 1 ordered Li_6_PS_5_Br (100/0) supercell with the positions of bromine (in red), sulfur (in yellow), and phosphorus (in green). The eight structures under investigation with corresponding labels are described below, where the percentages in parentheses indicate the distribution of bromine on the (4a/4d) sublattices, respectively. The values of configurational entropy are also provided for each structure. (b) Crystallographic sites suitable for lithium occupation, forming a distinct cage-like substructure around the Wyckoff 4d site, with the T5 lithium sites (Wyckoff 48h) in violet, T2 lithium sites (Wyckoff 48h) in orange, and the T4 lithium sites (Wyckoff 16e) in blue. T5a (Wyckoff 24g) positions are not depicted for clarity as they lie in between two T5. (c) Types of Li-ion jumps: intracage (T5–T5 doublet and T5–T2) and intercage (T5–T4–T5 and T2–T2).

Eight argyrodite structures Li_7−*x*_PS_6−*x*_Br_*x*_ were generated and characterized based on two key descriptors: the bromine occupation of 4a and 4d sublattices (Fig. 1a). Among these, six structures reflect the sublattice disorder, corresponding to experimentally determined site occupancies of sulfur and bromine.^41^ These specific structures were selected to correlate computational results with published experimental data. The structures include Li_6_PS_5_Br (88/12), Li_6_PS_5_Br (62/38), Li_5.7_PS_4.7_Br_1.3_ (88/37), Li_5.7_PS_4.7_Br_1.3_ (75/50), Li_5.5_PS_4.5_Br_1.5_ (88/62), and Li_5.5_PS_4.5_Br_1.5_ (75/75), where the percentages in parentheses indicate the distribution of bromine on the (4a/4d) sublattices, respectively. Since multiple orderings of S and Br in the sublattices can result in the same site occupancy, several unique configurations were optimized for each of the six structures (ESI Section A†). All further analyses were performed on the lowest energy configuration of each structure. Additionally, two configurations of Li_6_PS_5_Br exhibiting perfect order in the anionic sublattice, where bromine fully occupies 4a (100/0) or 4d (0/100) sites, were studied.

To measure disorder in the anionic sublattices, the configurational entropy (Δ*S*) was calculated using the sublattice model (eqn (1)).^42^ This model is universally applicable to crystalline materials and allows multiple sublattices to be considered (specifically the 4a and 4d sublattices in our study).1
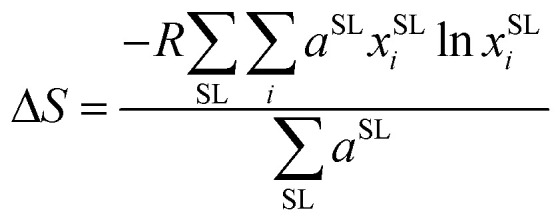
where *R* is the gas constant, *a*^SL^ is the number of sites on the SL sublattice (4a or 4d) and *x*^SL^_*i*_ is the fraction of element *i* randomly distributed on the SL sublattice.

In the latter part of the study, two more structures with the same cubic space group were created for additional analysis where both 4a and 4d sublattices are fully occupied either by sulfur (Li_7_PS_6_) or bromine (Li_5_PS_4_Br_2_).

### Density functional theory simulations

Density functional theory (DFT) calculations based on the Perdew–Burke–Ernzerhof functional for solid-state systems (PBEsol)^43,44^ within the Vienna *Ab initio* Software Package (VASP 6.3.2)^45^ were utilized. Projector augmented wave (PAW)^46^ potentials were used with cores of [He] for Li, [Ne] for P and S, and [Ar] for Br. Structure optimizations were conducted with an energy cutoff of 340 eV in 2 × 1 × 1 argyrodite supercells. The choice of a 2 × 1 × 1 supercell offers 8 Br and 8 S in the 4a and 4d sublattices (Wyckoff positions), respectively. This choice provides a suitable composition step size (Δ*x* = 0.125 in Li_7−*x*_PS_6−*x*_Br_*x*_) to study disorder at a moderate computational cost, close to the experimentally determined ratios. Nuclear magnetic resonance (NMR) shielding tensor calculations were performed in CASTEP utilizing the PBE functional^47^ and a 520 eV cutoff energy. Chemical shielding was calculated using the linear response method, translated into chemical shift values by comparison with reference compounds (Fig. S4 and Table S1†).^48,49^ To determine how Li site occupation affects chemical shift, calculations were performed in the primitive argyrodite cell for different Li positioning in the sublattice. Ultimately, we weighted each Li position-dependent signal based on the number of Li environments and the experimentally determined occupation for each structure.

### *Ab initio* molecular dynamics simulations

The ten lowest energy-optimized structures corresponding to different site disorders were subsequently studied with *ab initio* molecular dynamics (AIMD) in the canonical (NVT) ensemble using the Nosé–Hoover thermostat.^50,51^ The energy cutoff was reduced to 300 eV, and gamma-only *k*-point mesh was used. The selected time step was 2 fs for a total computational time of 150 ps. Macroscopic diffusion properties were obtained by performing multiple AIMD runs, covering a temperature range between 650 to 1000 K and fitting to Arrhenius behavior. Site-sensitive properties such as site occupancies, site-specific jump frequencies, and energy barriers were obtained using the analysis tools developed in our group.^52,53^ The AIMD simulation was separated into five parts to calculate the mean diffusion constants and standard errors.^54^ To analyze the individual jumps and occupancies, we defined three types of Li positions in the crystal lattice, namely, 48h (T5), 16e (T4), and 48h′ (T2),^55^ counting the times Li resides in these positions throughout the simulation, as well as the number of hops between these positions. Conductivity from rate-limiting jumps was calculated from AIMD simulations based on the jump frequency of rate-limiting jumps as previously described.^13^ To analyze the cage radius formed by lithium ions, the distances between diffusing cations and the closest anion at the 4a or 4d position were investigated and averaged within a 2 × 1 × 1 supercell using AIMD simulations at 300 K.

To analyze Li-ion jump activation energy specific to the local environments of sulfur and bromine, we examined AIMD simulations at 650 K for eight selected configurations of the Li_6_PS_5_Br composition. These configurations had different S and Br distributions on the 4a and 4d sublattices, encompassing every possible local environment for each type of jump. Local environment-specific activation energies for all eight configurations are presented in Table S2.†

We adopted a previously developed and described methodology for percolation analysis.^56^ Using the pymatgen library^57^ (version 2023.11.12), we generated structures of Li_6_PS_5_Br (100/0), Li_6_PS_5_Br (0/100), and Li_6_PS_5_Br (50/50) in a 5 × 5 × 5 supercell. For Li_6_PS_5_Br (50/50), twenty configurations with random arrangements of sulfur and bromine across the 4a and 4d sublattices were generated. For each configuration, the percolation model was applied with twenty iterations. In each iteration, an environment-specific activation energy was randomly selected within ±0.04 eV of the average values listed in Table S2.† This random selection accounts for the uncertainty in the activation energy values. The average results across all iterations and configurations per structure are analyzed.

The phonon density of states (DOS) can be calculated from the Fourier transform of the velocity autocorrelation function^58^ (VACF), which is defined as
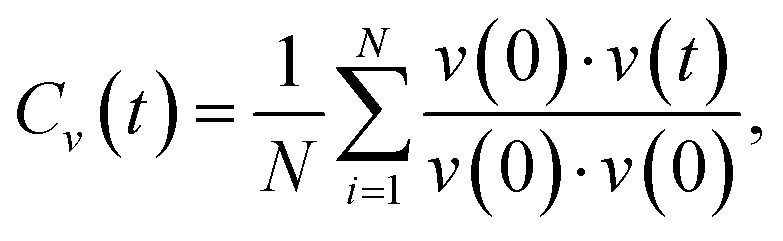
where *v*(*t*) is the velocity of an ion at time *t*. The indices *i* = 1…*N* indicate which ions the function is to be calculated over, allowing the phonon DOS to be projected over a subset of species in the trajectory. The band center of a projected phonon DOS is calculated as
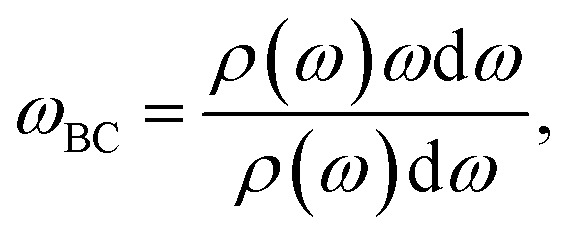
where *ω* is the phonon frequency and *ρ* is the phonon DOS at a given phonon frequency.^59^

The VACF is calculated from 300 K AIMD trajectories with at least 100 ps run time. Each VACF being split into three segments windowed with a Hann function^60^ and the phonon DOS is the average result across all three segments. All Fourier transforms and windowing is handled using the signal processing capabilities in SciPy.^61^

### Data analysis

We compiled a comprehensive dataset (Table S3†) of measured conductivity values and elemental occupations at the 4a and 4d sublattices for argyrodites and their derivatives, specifically those denoted as Li_7−*x*_ACh_6−*x*_X_*x*_ (A = P, Si, Cu, Sb; Ch = S, Se, O; X = Cl, Br, I, CN). The dataset was manually collected from available literature sources. Data points were selected, ensuring that each entry included the specific argyrodite composition, measured conductivity, and occupations of the 4a and 4d sublattices. Entries without complete crystallographic data (occupations at both 4a and 4d sites) were excluded to maintain dataset integrity.

The average ionic potential within the sublattice was calculated using the following equation:2
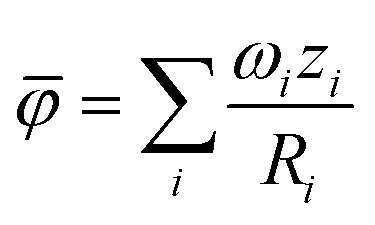
where *ω*_*i*_ is the amount of anion *i* with charge *z*_*i*_ and ionic radius *R*_*i*_. Ionic radius values were extracted from ref. 62 and 63, considering the coordination and oxidation state of the element.

For the correlation analysis, the min–max normalization method was applied to the descriptors: average ionic potential of the 4d (*φ̄*_4d_) and 4a (*φ̄*_4a_) sublattices and absolute deviation of the average ionic potentials ratio from one 
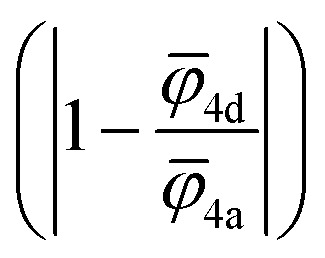
. Both Pearson's (*R*_Pearson_) and Spearman's rank (*R*_Spearman_) correlation coefficients were calculated between conductivity and the corresponding descriptors, with significance levels indicated. For further analysis, conductivity was modeled as an exponential function of a linear combination of all three descriptors 
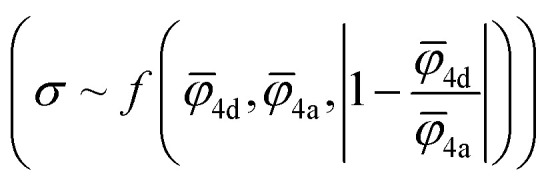
 and was fitted using the SciPy^61^ library (version 1.11.2).

## Results and discussion

Lithium argyrodite Li_6_PS_5_Br crystallizes in the cubic *F*4̄3*m* space group (216). In the ordered crystal structure, halide ions are located at the Wyckoff 4a positions, while S^2−^ ions (not bonded to P^5+^) occupy the Wyckoff 4d positions (Fig. 1a). Halide and sulfur ions can exhibit site disorder by exchanging positions and occupying both 4a and 4d sites. The anion framework forms 136 interstitial tetrahedral voids per unit cell, suitable for cation occupancy. Four of these voids are filled by P^5+^ cations at the Wyckoff 4b site, forming PS_4_^3−^ tetrahedra. The remaining 132 tetrahedral voids can accommodate lithium.^55^ Lithium ions distributed across T5 (Wyckoff 48h) positions form a cage-like substructure encircling the 4d site (Fig. 1b). Earlier research categorized Li^+^ ion positions into T5 (Wyckoff 48h), T5a (Wyckoff 24g), and T2 (Wyckoff 48h),^12,13,21,64,65^ defining three types of Li-ion jumps: doublet, intracage, and intercage (Fig. 1c). Doublet and intracage jumps represent short-range movements within a cage engaging T5 and T2 sites, while long-range intercage jumps involve T2–T2 transitions, linking adjacent cages.^12,64^ Moreover, an additional pathway facilitating intercage jumps through the interstitial T4 site, positioned between cages, has been identified (T5–T4–T5).^12,23,25,30^ While all three jump types contribute significantly to the rapid diffusion of lithium ions, the intercage jump is typically considered as the rate-limiting step.^13,21^

To study the impact of site disorder (S^2−^/Br^−^), we generated eight argyrodite Li_6−*x*_PS_5−*x*_Br_1+*x*_ (*x* = 0, 0.3, 0.5) structures labeled by percentage of bromine occupation across 4a and 4d positions, as depicted in Fig. 1a. The selection of structures was made based on a recent set of experimentally characterized data^41^ that provides a diverse distribution and composition of halogen and allows us to validate results of AIMD simulations. Thus, six structures reflect the sublattice disorder, incorporating experimentally determined S and Br site occupancies.^41^ Additionally, we included Li_6_PS_5_Br exhibiting perfect order in the anionic sublattice, where bromine fully occupies either 4a (100/0) or 4d (0/100) sites. Even though both perfect-ordered structures have not been obtained experimentally to date, including them expands our dataset, allowing for a more comprehensive understanding of the effects of S^2−^/X^−^ disorder on the structure and ionic transport within the argyrodite framework.

ESI Section A† details the structural characterization of the argyrodite structures, as outlined in the Computational details section. This analysis is crucial to verify how well our models correspond with experimental structural trends. The ESI† provides calculated lattice parameters, Li-ion cage sizes, and computed Nuclear Magnetic Resonance (NMR) spectroscopy parameters that closely align with experimental trends and enhance our understanding of the mechanisms involved.

### Influence of configurational entropy and total bromine content on Li-ion transport

Halogen-rich lithium argyrodites have demonstrated a significant enhancement in conductivity compared to Li_6_PS_5_X (X = Cl, Br, I) compositions.^41,66,67^ The substitution of sulfur with halogen anions enhances intercage transport by weakening the electrostatic interaction between lithium and anions^23,24^ and introducing more lithium vacancies into the lattice.^18,21^ In addition, halide doping tends to soften the anionic lattice, substantially reducing the activation barrier.^24,25^ Further studies on the influence of anion disorder in halogen-rich argyrodites established a link between structural complexity and ionic diffusion, proposing that tailoring configurational entropy is a potential strategy for developing highly conductive materials.^37^ Distinguishing between the influences of increased halide content and anionic site disorder presents a challenge due to their interconnected nature. In this section, we aim to validate the correlation between lithium transport and both total Br content and configurational entropy at the anion sublattice, which are believed to be the primary determining factors of conductivity in argyrodites.

**Fig. 2:**
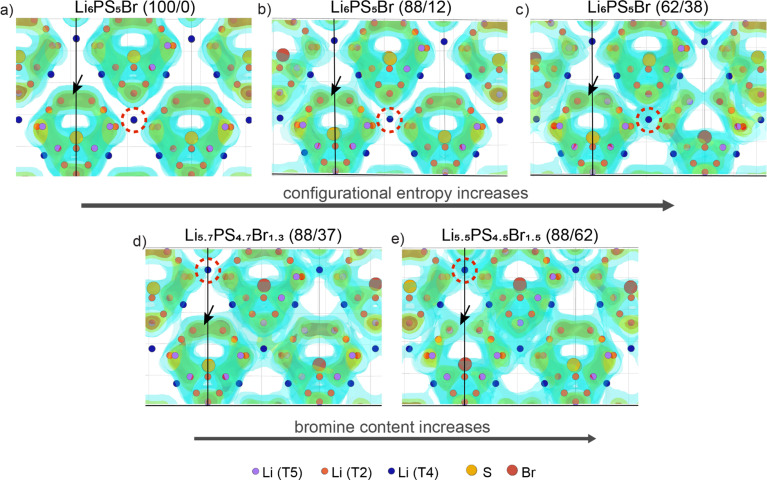
Probability density of Li^+^ obtained from AIMD simulations performed at 650 K. The area encircled by the red dashed line indicates an increase in lithium density between adjacent cages, while the black arrow highlights a decrease in lithium density for the intracage transport. The structures presented are: (a) Li_6_PS_5_Br (100/0), Δ*S* = 0.00 *R*; (b) Li_6_PS_5_Br (88/12), Δ*S* = 0.35 *R*; (c) Li_6_PS_5_Br (62/38), Δ*S* = 0.66 *R*; (d) Li_5.7_PS_4.7_Br_1.3_ (88/37), Δ*S* = 0.52 *R*; (e) Li_5.5_PS_4.5_Br_1.5_ (88/62), Δ*S* = 0.51 *R*.

To illustrate the Li^+^ migration pathways through the bulk structure, we extracted lithium probability density distributions from AIMD simulations. Fig. 2a–c showcase the effect of increased configurational entropy at the anion sublattice in the Li_6_PS_5_Br structures, while Fig. 2d and e demonstrate the impact of increased bromine content within structures having a similar configurational entropy. Comprehensive Li^+^ distribution plots for all examined structures are available in Fig. S5.†

For the examined structures, both an increase in configurational entropy and higher bromine content reveal a similar pattern of lithium redistribution associated with enhanced intercage diffusion and a comparatively flatter energy landscape, contrasting to the more distinct density profile observed in the ordered Li_6_PS_5_Br. In particular, the Li_6_PS_5_Br (100/0) structure with Δ*S* = 0.00 *R* shows Li-ion density concentrated within isolated cages formed by doublet and intracage jumps with no diffusion between cages (Fig. 2a). As configurational entropy increases, regions of high Li-ion density become interconnected with neighboring cages, indicating intercage diffusion. This is evident from the increasing lithium density encircled by the red dashed line (Fig. 2). However, it is worth noting that as the occurrence of intercage diffusion increases, the frequency of doublet jumps decreases, as indicated by a decrease in Li density within cages (highlighted by a black arrow in Fig. 2).

These observations are supported by the analysis of mean squared displacements (MSDs) extracted from our AIMD simulations within the same simulation time frame (Fig. S6†). The distance for intracage diffusion measures around 4.5 Å (ref. 26), while intercage diffusion covers approximately 7 Å (ref. 13). Therefore, if doublet and intracage jumps dominate, the expected MSD would be around 4.5^2^ ≈ 20 Å^2^, while intercage jumps would correspond to a larger MSD of approximately 7^2^ ≈ 50 Å^2^. For the ordered Li_6_PS_5_Br (100/0) structure, jumps occur only within the cage at lower temperatures, as indicated by MSD values below 50 Å^2^ (Fig. S6a†). In contrast, both increased configurational entropy and higher bromine content enable diffusion even at lower temperatures, resulting in MSD values above 50 Å^2^, corresponding to long-range transport.

Our observations indicate that improved lithium diffusion can be achieved through both an increase in configurational entropy at the anion sublattice and higher bromine content. Further, our phonon density of states (DOS) calculations, as illustrated in Fig. S7,† show that both of these optimization approaches contribute to the softening of the lattice, thereby enhancing lithium ion movement. Notably, increased bromine content without a change in configurational entropy, as well as higher configurational entropy within the same composition, both lead to better intercage diffusion and reduced intracage transport. In other words, two structures with the same configurational entropy or bromine content can still exhibit vastly different transport properties, underscoring a nonlinear relationship. Such findings suggest a more complex dependency between enhanced conductivity and these two factors, contrasting with the straightforward correlation often proposed in the literature.^23,28,37^

### Bromine occupation at 4d as a key descriptor for Li-ion transport

Sulfur/halide site disorder has been extensively studied in argyrodite materials (Li_6−*x*_PS_5−*x*_(Cl, Br, I)_1+*x*_) and, similar to configurational entropy and total bromine content, is often considered a key factor for lowering the activation barrier and promoting conductivity.^13,17–20,23,24,26^ The halide occupancy at the 4d site is typically denoted “site disorder”, which can be confusing because when there is 100% site disorder — where unbonded S^2−^ exclusively occupies the 4a sites and the halogen occupies the 4d sites — there is actually no site disorder present. Moreover, for halogen-rich compositions, halide occupancy only at the 4d site does not directly reflect site disorder.

**Fig. 3:**
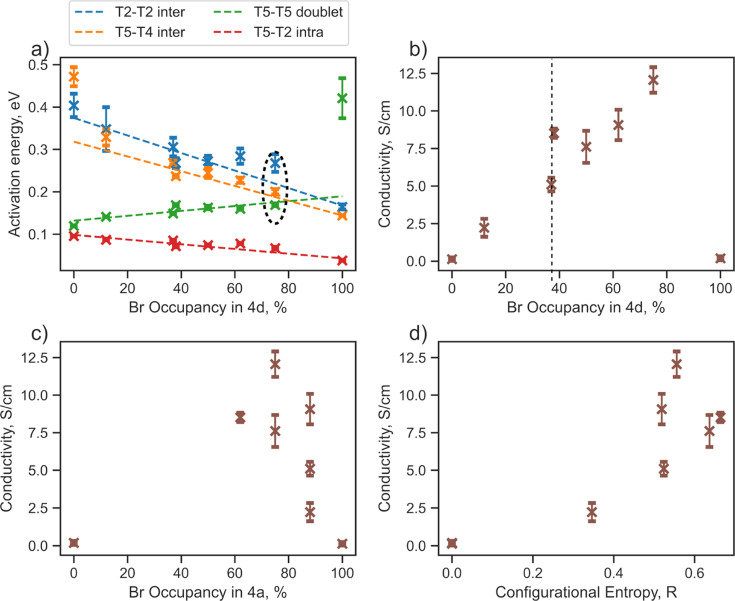
Relationship between discussed descriptors and diffusion properties extracted from jump analysis performed by tracking lithium jumps (starting and ending positions) for Li_6−*x*_PS_5−*x*_Br_1+*x*_ structures during AIMD simulations at 650 K. (a) Activation energies per jump type depending on bromine occupancy in the 4d site. (b) Relationship between conductivity, calculated from rate-limiting jumps, and bromine occupancy in the 4d site. (c) Relationship between conductivity, calculated from rate-limiting jumps, and bromine occupancy in the 4a site. (d) Relationship between conductivity, calculated from rate-limiting jumps, and anion configurational entropy.

The next part of our research will focus on lithium jump analysis considering bromine occupancy at the 4d and 4a sites, as well as configurational entropy, as critical descriptors for the diffusion properties of argyrodite materials (Fig. 3). Our observations indicate that the activation energy for intercage jumps tends to decrease with more bromine occupying the 4d site. In contrast, the activation energy for doublet jumps increases, and when the 4d site is fully occupied by bromine, the T5–T5 doublet jump becomes rate-limiting (Fig. 3a). These trends support findings from previous studies,^13,20^ assuming that the overall rate of Li diffusion is determined by intercage jumps in structures with low Br-occupancy at 4d sites and by doublet jumps when high Br-occupancy is present.

Specifically, the Li_6_PS_5_Br (0/100) structure shows Li-ion probability density concentrated within isolated cages; however, contrary to Li_6_PS_5_Br (100/0) structure discussed above, lithium ions are concentrated around the 4a sites rather than the 4d positions (Fig. S5b†). This is due to the stronger attraction between lithium cations and sulfur compared to halide anions,^2,20,68,69^ which forces lithium to create new cages surrounding the 4a sites, fully occupied by sulfur in this case. Regarding the new 4a cage, the former intercage jump becomes intracage, while the former T5–T5 doublet (intracage) becomes a new intercage pathway, explaining the switch of rate-limiting step from intercage jump to a doublet with increased bromine occupation at 4d site. For clarity and to avoid further confusion, we will continue using the former nomenclature of jumps.

The ionic conductivity calculated based on rate-limiting jumps is presented in Fig. 3b–d and S8.† Both ordered structures, Li_6_PS_5_Br (100/0) and Li_6_PS_5_Br (0/100), exhibit low values of conductivity, aligning with the outcomes from the Li probability density (Fig. S5†) and MSD (Fig. S6†) analyses. Interestingly, the conductivity trend does not follow a straightforward monotonic relationship with bromine occupancy in the 4d (Fig. 3b), 4a (Fig. 3c) sites or configurational entropy (Fig. 3d).

For example, the structures Li_6_PS_5_Br (62/38) with Δ*S* = 0.66 *R* and Li_5.7_PS_4.7_Br_1.3_ (88/37) with Δ*S* = 0.52 *R*, despite having similar bromine occupancies of around 40% at the 4d site, exhibit distinct differences in conductivity values (Fig. 3b, highlighted by a dashed line). A noteworthy distinction between these structures is their bromine occupancy at the 4a site and configurational entropy values. Another example is the Li_5.5_PS_4.5_Br_1.5_ (75/75) structure with Δ*S* = 0.55 *R*, which has the highest conductivity among the investigated structures and demonstrates remarkably similar activation energy values for the T5–T5 doublet jump, and both intercage jumps (Fig. 3a, encircled with black dashed lines). However, this conductivity maximum does not align with the maximum bromine occupancy in one of the sublattices or configurational entropy (Fig. 3b–d). These observations underscore the complexity of the factors influencing conductivity in argyrodite materials, indicating that neither bromine occupancy at the 4d, 4a sites nor configurational entropy alone can accurately explain the conductivity trend.

To further investigate the impact of sulfur/bromine distribution across 4a and 4d sites on lithium diffusion, we will focus on the cages formed by Li ions around the 4a and 4d positions. The formation of these cages causes significant changes in rate-limiting jumps and activation energies.

**Fig. 4:**
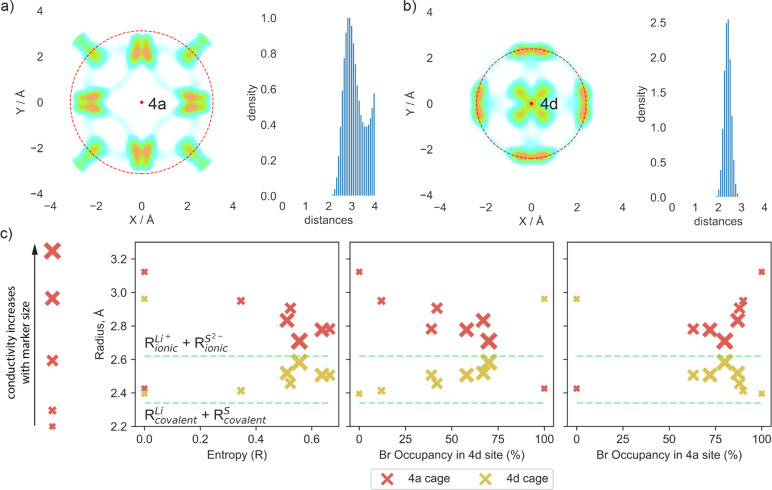
Comparison of radii of both cages centered at 4d and 4a sites. The radius of the cage is calculated as the average distance between the lithium positions and cage centers within a 2 × 1 × 1 supercell during the AIMD simulation at 300 K with a 4 Å cutoff. Cages with lithium probability density around are shown in XY projection. The red dashed line highlights the average radius. (a) Example of an individual lithium cage centered at the 4a site (red dot on the plot) shown in XY projection, coupled with the distribution of distances between lithium and the cage center for Li_6_PS_5_Br (100/0) structure. (b) Example of an individual lithium cage centered at the 4d site (red dot on the plot) shown in XY projection, coupled with the distribution of distances between lithium and the cage center for Li_6_PS_5_Br (100/0) structure. (c) Relationship between average cage radii for all investigated structures with entropy and bromine occupancy at both sublattices. Red markers are related to the cage surrounding the 4a site, and yellow markers represent the cage centered at the 4d site. Thus, each material is characterized by two markers. The markers' size is linked to the material's conductivity; the bigger the marker, the higher the conductivity.

To quantify the changes in lithium transport and interactions with the anion framework caused by differences in sulfur/bromine distribution across 4a and 4d sites, we measured the average distance between cage centers (4a and 4d) and lithium positions, expressed as a cage radius (Fig. 4). We observe an expansion of 4d cages due to higher bromine occupancy in 4d sites (Fig. 4c), which shortens the distance for intercage jumps. However, simultaneously, the distance for doublet jumps increases, leading to higher activation energy and making doublet jumps the rate-limiting step with excessively high bromine content in the 4d site. The opposite trend is observed for the radius of the cage centered at the 4a sites.

Analysis of cage radii presents a different way of expressing the impact of S^2−^/Br^−^ disorder across the 4d and 4a sites. A high degree of disorder, and thus a high configurational entropy on the anion sublattice makes the radii of the 4a and 4d cages similar, resulting in a more uniform distribution of electrostatic forces and smoothing the path for lithium ions. In the extreme cases with no S^2−^/Br^−^ disorder, such as in Li_6_PS_5_Br (100/0) and (0/100), the electrostatic forces trap the lithium ions near sulfur-occupied sites, limiting their diffusion as demonstrated by lithium probability density analysis (Fig. S5a and b†). It is also worth noting that in these cases, with no S^2−^/Br^−^ disorder, the radii of cages formed by lithium around sites fully occupied by sulfur are close to the sum of lithium and sulfur covalent radii (Fig. 4c), suggesting a much stronger interaction between sulfur and lithium − positioned between covalent and ionic − which consequently resists lithium long-range transport. Previous research, such as the study of Li_3_InBr_6_ (ref. 70), showcased that the mixed ionic–covalent interaction and lattice frustration between ionic and covalent bonding preferences contribute to a more favorable energy landscape for ion conduction. Similarly, in argyrodite structures, S^2−^/Br^−^ disorder within a sublattice modulates the bond strength between lithium and the anionic framework, facilitating faster bulk diffusion.

As discussed previously, the maximum lithium ionic conductivity does not align with the maximum anion configurational entropy or the maximum bromine occupancy at one of the sublattices. Instead, the highest conductivity occurs in the structure where the 4a and 4d cages have nearly the same radius (Fig. 4c). The structure Li_5.5_PS_4.5_Br_1.5_ (75/75) exhibits an equal distribution of S^2−^ and Br^−^ across both 4a and 4d sublattices, resulting in similar radii of lithium cages formed around the 4a and 4d sites (Fig. 4c). This leads to comparable activation energies for intra- and intercage movements (Fig. 3a) and high conductivity observed in both AIMD simulations and experimental measurements. This observation emphasizes the significance of achieving a balanced S^2−^/Br^−^ disorder across both sublattices, rather than simply maximizing the bromine content at the 4d site or the configurational entropy, in optimizing long-range lithium transport in argyrodite materials. Our findings align with observed correlations between uniformity in inter- and intracage jump distances,^36,71^ as well as in the sizes of the 4a and 4d cages,^68,69,72^ with improved ionic conductivity. It is also worth noting that the distribution of S^2−^ and Br^−^ across the 4a and 4d sublattices in argyrodites can be tuned not only by halide doping techniques but also through compositional changes or synthetic conditions. For example, aliovalent substitution of phosphorus has been shown to affect S^2−^/I^−^ disorder,^15^ while quenching in liquid nitrogen during synthesis can be used to vary sulfur/halide disorder in argyrodites.^26^

### Design strategies for argyrodite conductors

The analysis presented above highlights the pivotal role of the distribution of S^2−^ and Br^−^ ions across the 4a and 4d sublattices in defining the properties of argyrodite materials. This section aims to formulate a descriptor based on the above observations, guiding the design of new argyrodite materials through tailoring the structural disorder, aiming at increasing the conductivity. To realize this, we analyze a broad range of argyrodite compositions, primarily focusing on the 4a and 4d anionic sublattices.

**Fig. 5:**
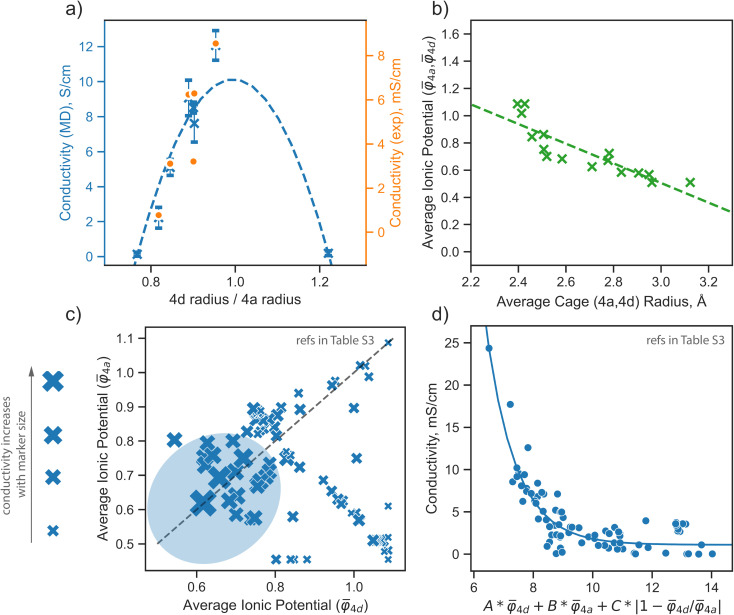
Design strategies for argyrodite materials employing average ionic potentials within the anion sublattices. (a) Relationship between the ratio of 4d to 4a average cage radii and their corresponding conductivity values, derived from rate-limiting jumps observed in AIMD simulations at 650 K and experimental data.^41^ (b) Correlation between average cage radii centered in 4d and 4a sites extracted from AIMD and the average ionic potentials within these sublattices calculated using eqn (2). (c) Comparison of average ionic potentials between 4a and 4d sublattices in experimentally synthesized structures (Table S3†), with marker size indicating measured conductivity values. (d) Correlation of conductivity as a function of average ionic potentials across both sublattices 
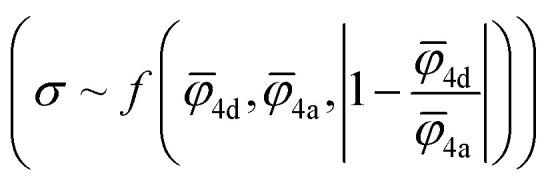
. The function *f* (represented by a solid line) models conductivity as an exponential function of a linear combination of 
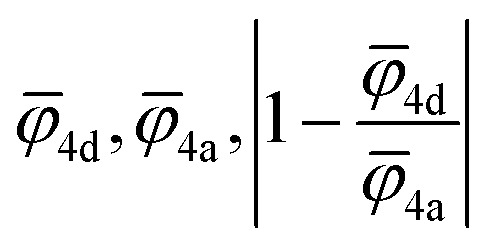
 values. The Pearson correlation coefficient for the fitted function is *R*_Pearson_ = 0.90 (*p* < 0.001).

In the argyrodite structures we examined, specifically Li_6−*x*_PS_5−*x*_Br_1+*x*_, the maximum lithium ionic conductivity is observed when the average distances between lithium ions and anions distributed across the 4a and 4d sublattices are nearly equal, as discussed in the previous paragraph. This trend is consistent with conductivity values calculated from AIMD simulations and those obtained from experimental measurements^41^ (Fig. 5a). While AIMD simulations and some computational methods can be employed to calculate cage radii, they are insufficient for the rapid prescreening needed for materials design.

Achieving a homogeneous distribution of S^2−^ and Br^−^optimizes the electrostatic environment between lithium and the anion framework, which is crucial for diffusion in argyrodites.^12,22,28,30,34–36^ To compare the strength of attraction between the diffusing cation and the anions located within the 4a and 4d sites, we employed the ionic potential^73^ as a simple descriptor to explain lithium transport in argyrodites. This metric has been successfully used in the literature to describe the properties of various materials.^74–79^ The ionic potential is defined as the ratio of ionic charge to ionic radius, reflecting the charge density at an ion's surface, capturing both electrostatic bond strength and steric effects.

The average ionic potential, calculated within a sublattice using eqn (2), demonstrates a linear correlation with cage radii calculated from AIMD simulations (Fig. 5b), highlighting its ability to reflect changes in the structural and electrostatic environment influencing lithium transport.

To further explore the utility of ionic potential in understanding and designing argyrodite materials, we collected a dataset encompassing a broad range of argyrodites, denoted as Li_7−*x*_ACh_6−*x*_X_*x*_ (A = P, Si, Cu, Sb; Ch = S, Se, O; X = Cl, Br, I, CN), as described in Computational details. This dataset includes experimentally measured conductivity values and elemental occupations at the 4a and 4d sublattices (Table S3†). To compare ionic potentials within both sublattices, we plotted the calculated average ionic potentials of the 4a and 4d sublattices against each other (Fig. 5c), with marker sizes corresponding to conductivity values. The experimental data indicates that the highest conductivity is generally observed in regions where both sublattices display lower, nearly identical ionic potentials, identified by a blue area in the plot (Fig. 5c).

To understand how each factor individually influences conductivity, we explore the relationship between conductivity and parameters, such as the average ionic potentials of the 4d and 4a sublattices and their ratio (Fig. S9†). The correlation between conductivity and the average ionic potential in the 4d sites suggests that materials with lower average ionic potential in the 4d sublattice exhibit higher conductivity. The correlation between conductivity and the average ionic potential in the 4a sublattice is less pronounced. Given the inherently smaller size of the 4d cage compared to the 4a cage,^72^ variations in the 4d sublattice, particularly its tendency to expand, significantly influence conductivity. The influence of the ratio of both potentials shows that minor deviations from equality are associated with significant impacts on conductivity. Once these deviations exceed a certain threshold, they no longer affect conductivity substantially. The correlation coefficients between conductivity and individual descriptors were not exceptionally high, underscoring the need to consider all three parameters together rather than relying on individual descriptors. An observed robust exponential relationship between conductivity and a linear combination of the average ionic potentials of both sublattices along with their ratio (Fig. 5d), supports this conclusion.

Our results underscore that reducing the ionic potential equally on both sublattices (4a and 4d) greatly enhances conductivity. Reduced average ionic potential weakens coulombic interactions between the anionic sublattice and the diffusing ion, while a nearly equal ratio of ionic potentials within both sublattices suggests a homogeneous electrostatic environment prone to rapid lithium transport. In argyrodite structures, sulfur in the 4d position has a high ionic potential, presenting an opportunity for improvement. Isovalent substitution of sulfur with atoms with a larger ionic radius^23,33^ or aliovalent substitution with a higher amount of halogen atoms with lower ionic potentials enhances conductivity. For example, high conductivity has been forecasted for Li_5_PS_4_X_2_ and Na_5_PS_4_X_2_ (X = Cl, Br, I) structures by computational studies.^13,54,71^ Although Li_5_PS_4_X_2_ has not yet been synthesized, Li_5.3_PS_4.3_X_1.7_ structures demonstrated increased conductivity in experiments.^17,20,24^ Further improvement can be achieved by a homogeneous distribution of halides across 4a and 4d sites. Structures with an equal distribution of halides have been shown to be the most conductive argyrodites to date^23,24,37,41,67^^.^

### Role of sulfur/bromine local environments in argyrodite materials

To complement the design criteria and deepen our understanding of how the anionic arrangement within sublattices affects lithium-ion transport, we analyzed the local environments created by anionic distribution and their impact on the activation energy for lithium ion jumps. To simplify the analysis, we focused only on the jumps through the T5 and T4 sites, as T5–T4–T5 and T5–T5 doublet jumps are pivotal in lithium diffusion.

**Fig. 6:**
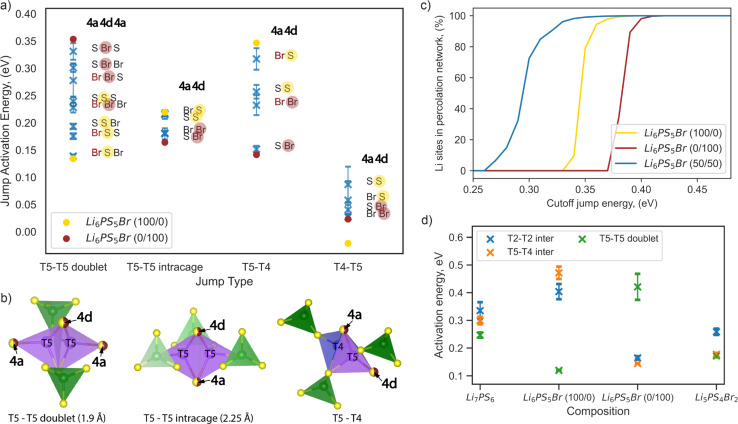
Impact of sulfur/bromine local environments on jump activation energy. (a) Comparison of jump activation energies per possible jump environment within Li_6_PS_5_Br composition, calculated from AIMD at 650 K. Blue markers show the average jump activation energy with error bars representing variations across eight Li_6_PS_5_Br 2 × 1 × 1 supercells with different site disorder (Table S2†). Yellow and red solid dot markers indicate the jump activation energies of Li_6_PS_5_Br (100/0) and Li_6_PS_5_Br (0/100), respectively. Sulfur and bromine occupancy in the 4d position is highlighted by yellow and red half-transparent circles, respectively. (b) Illustration of the coordination of T5 and T4 tetrahedra for each jump type, where the 4a and 4d sites used for jump environment nomenclature are shown as partially occupied by sulfur and bromine (half-yellow, half-red dots) and highlighted by arrows. The T5 tetrahedra (in violet) are formed by two S^2−^ ions (corner-shared with PS_4_ tetrahedra in green) and two anions at the 4a and 4d sites, respectively. T4 sites (in blue) are coordinated by three S^2−^ ions (also corner-shared with PS_4_ tetrahedra in green) and one anion at the 4a site. (c) Energy-percolation diagram showing the fraction of Li sites connected to a percolating network for Li_6_PS_5_Br (100/0), Li_6_PS_5_Br (0/100), and Li_6_PS_5_Br (50/50) (averaged over twenty 5 × 5 × 5 supercells), as detailed in the Computational details section. (d) Comparison of activation energies per jump type in Li_7_PS_6_, Li_6_PS_5_Br (100/0), Li_6_PS_5_Br (0/100), and Li_5_PS_4_Br_2_, as determined from AIMD simulations at 650 K.

We investigated the impact of the type of anion at the 4a and 4d sites on the Li-ion jump activation energy by examining AIMD of eight configurations of the Li_6_PS_5_Br composition having different S and Br distributions on these sublattices (Table S2†). The structure set was chosen to ensure multiple repetitions of each possible anionic environment and to obtain reliable statistics. The average activation energy for each type of jump across the different local environments is depicted in Fig. 6a. We characterized a jump environment by labeling the anions occupying the 4a and 4d positions of the start site and the 4a position of the end site in T5–T5 doublet jumps, as both T5 sites share the same 4d position but differ in 4a positions (Fig. 6b). For T5–T5 intracage, T5–T4, and T4–T5 jumps, the labels include the anions occupying the 4a and 4d positions since both the start and the end sites share the same environment (Fig. 6b).

Examining the Li_6_PS_5_Br (100/0) and (0/100) structures without site disorder, we found that the activation energies for the T5–T4 and T5–T5 doublet jumps are critical limiting factors, as indicated in Fig. 6a and previously in Fig. 3a. To enhance ion conductivity in argyrodites, our goal is to minimize the activation energies for these pivotal jumps, which typically follow contrasting trends. Specifically, the activation energy of the T5–T5 doublet jump tends to be lower when sulfur occupies the 4d position, while a lower activation energy for the T5–T4 jump is observed when bromine occupies the 4d position (Fig. 6a).

The correlation between jump activation energy and 4d site occupation is more direct compared to the 4a site occupation. For each jump type, the environments with the 4d site consistently occupied by the same element — either sulfur (yellow half-transparent circles) or bromine (red half-transparent circles) — are generally grouped together by activation energy value (Fig. 6a). The effect of the 4a site occupation can be further deconvoluted. For example, bromine occupation in the 4a site (red letters) lowers the jump activation energy for T5–T5 doublet jumps while increasing the jump activation energy for T5–T4 jumps, maintaining the same occupation in the 4d site (Fig. 6a). This observation is consistent with our previous analysis of the impact of ionic potentials (Fig. S9†), highlighting the differential roles of the 4d and 4a sublattices in influencing lithium transport dynamics.

Our results suggest that mixed occupation of 4d and 4a sites by both sulfur and bromine, introduced by site disorder, creates low-energy local environments that facilitate lithium transport. However, attempts to maximize the low-energy local environment for one type of rate-limiting jump simultaneously create high-energy environments for another. For example, the “Br S S” and “S S Br” environments lower the activation energy of T5–T5 doublet jumps but create high-energy “Br S” and “S S” environments for T5–T4 jumps (Fig. 6a). Therefore, an even distribution of high- and low-energy environments for both rate-limiting jumps should be beneficial for long-range lithium diffusion.

To test this hypothesis, we employed a percolation model, which has been previously introduced and can be applied to disordered solid electrolytes.^56^ We created structures of Li_6_PS_5_Br (100/0) and Li_6_PS_5_Br (0/100) without site disorder, as well as Li_6_PS_5_Br (50/50) with bromine equally distributed across the 4a and 4d sites, in a 5 × 5 × 5 supercell. For Li_6_PS_5_Br (50/50), twenty randomly generated distributions of sulfur and bromine across 4a and 4d positions were analyzed to obtain reliable statistics, as detailed in the Computational details section. Our analysis indicates that, on average, each local environment across jump types appears with equal probability for Li_6_PS_5_Br (50/50) (Fig. S10†). In the percolation model, a connection between two lithium sites is considered to exist if the local environment-specific activation energy for both the forward and backward jumps is below a predefined cutoff energy value. When a connected path spanning the entire length of a supercell can be found for a given cutoff jump energy value, this path is termed “percolating,” ensuring that the endpoint of the percolation also serves as a starting point for the percolating path.

Fig. 6c shows the fraction of lithium sites connected to a percolating network as a function of cutoff energy value. For both ordered structures Li_6_PS_5_Br (100/0) and Li_6_PS_5_Br (0/100), percolation becomes possible only with cutoff energy values higher than the corresponding average activation energy of rate-limiting jumps of 0.32 ± 0.04 and 0.33 ± 0.04 eV, respectively (Table S2†). In contrast, for the Li_6_PS_5_Br (50/50) structure, percolation is possible with a cutoff energy higher than 0.26 eV (Fig. 6c), where all types of jumps (T5–T5 doublet, T5–T5 intracage, T5–T4, and T4–T5) are available according to local environment-specific activation energy (Fig. 6a). The findings from the percolation model suggest that S^2−^/Br^−^ disorder across both sublattices enables percolation through Li_6_PS_5_Br by creating a variety of local environments for lithium transport. While both low-energy and high-energy local environments appeared, equal distribution of bromine across 4a and 4d sites allows for a lower average activation energy of percolation.

While evenly mixed environments are beneficial, this is not the only solution for enhancing lithium diffusion. Interestingly, for homogeneous environments (where both 4a and 4d sites are occupied by only sulfur or only bromine), the activation energies are comparable for T5–T5 doublet (“S S S” and “Br Br Br”) and T5–T4 (“S S” and “Br Br”) jumps (Fig. 6a). Structures with an increased number of these homogeneous local environments could enhance lithium diffusion due to fewer high-energy T5–T5 doublet and T5–T4 jumps. To test this hypothesis, we constructed and analyzed Li_5_PS_4_Br_2_ and Li_7_PS_6_ structures *via* AIMD, maintaining the same cubic space group. As predicted, our results showed no significant difference between jump activation energies in Li_5_PS_4_Br_2_, making it challenging to identify a single rate-limiting step (Fig. 6c). The same behavior was observed for Li_7_PS_6_. Consistent with our previous discussion, Li_5_PS_4_Br_2_ shows lower average activation energies compared to Li_7_PS_6_, attributable to its reduced ionic potential, which decreases the coulombic interactions between the anion sublattices and the Li-ions. Increased vacancy concentration in Li_5_PS_4_Br_2_ may also contribute to the observed reduction in activation energies. Our findings align with prior research suggesting that the substitution of sulfur with halogen atoms, as well as the reverse process (substitution of halogen with sulfur), enhances the MSD values and ionic mobility.^16^ However, this increase is more restrained in sulfur-rich structures due to strong Li–S interactions, which limit the kinetic freedom of lithium ions.

## Conclusion

This study investigates the mechanisms behind fast ionic conductivity in Li_7−*x*_PS_6−*x*_Br_*x*_ argyrodites, utilizing *ab initio* molecular dynamics to examine the impact of the S^2−^/Br^−^ local arrangement. Our findings indicate that the increased configurational entropy and bromine content cause a redistribution of lithium probability density, enhancing diffusion by creating a more uniform energy landscape than in anion-ordered configurations. However, contrary to what has often been reported in the literature, lithium ionic conductivity does not exhibit a straightforward correlation with bromine content, its occupancy at the 4d site, or configurational entropy. Instead, the maximum conductivity is observed in structures where bromine and sulfur are evenly distributed across the 4a and 4d sublattices, leading to similar sizes in lithium 4a- and 4d-centered cages, facilitating similar jump activation energies and distances between lithium and both anionic sublattices.

Our analysis suggests that both intercage and doublet jumps are equally crucial for lithium transport. The inversion of sulfur and bromine in the sublattices triggers a redistribution of lithium, forming new sulfur-centered cages and shifting the rate-limiting step from intercage to doublet jumps. This shift is driven by the stronger attraction between lithium cations and sulfur compared to bromine anions.

To guide the design of argyrodite materials, we introduce the ionic potential, which reflects the charge density at an ion's surface, as a simple and universal descriptor. This descriptor assesses the strength of attraction between diffusing cations and anions within the 4a and 4d sublattices. A thorough analysis of experimental data on argyrodite conductivities demonstrates that the ionic potential effectively captures changes in cage radii formed by Li-ion around 4a or 4d sites, thereby serving as a reliable estimator of argyrodite conductivity. Our study proposes that maximum conductivity can be achieved by minimizing the average ionic potentials on the 4d and 4a sublattices while ensuring that both values remain equal.

Further analysis of local sulfur/bromine environments and their impact on activation energies for lithium jumps revealed that site disorder in 4a and 4d positions creates low-energy paths for intercage diffusion while simultaneously introducing high-energy environments for doublet jumps. The average energy for lithium percolation can be reduced by an even distribution of high- and low-energy environments for both rate-limiting jumps, achievable through an equal distribution of sulfur and bromine across both 4a and 4d sites. Additionally, homogeneous environments with only bromine or sulfur occupying both 4a and 4d sites could lower the average percolation energy by reducing the number of high-energy environments for intercage and doublet jumps, achieving similar activation energies. This analysis supports outcomes from the ionic potential design criteria, underscoring the critical role of equal anionic distribution in optimizing the conductivity of argyrodites over merely maximizing bromine content, 4d site occupancy, or configurational entropy.

Overall, our work deconvolutes the factors influencing ionic conductivity in argyrodite materials, detailing the impact of site disorder and local sulfur/bromine environments while introducing descriptors that unravel observed experimental trends. We provide a unified, in-depth understanding of how atomic arrangements affect ion mobility, marking a significant advancement in optimizing electrolyte design for energy storage solutions.

## Data availability

The data supporting this article have been included as part of the ESI.† Additional computational data produced in this work is available from the corresponding authors upon reasonable request.

## Author contributions

A. K. L. and A. V. wrote the paper with contributions from all co-authors. A. K. L. J. A. Q. and A. V. performed DFT and MD calculations. M. W. and A. V. designed the work.

## Conflicts of interest

There are no conflicts to declare.

## Supplementary Material

TA-012-D4TA04628E-s001
