# Supplementary material for: Optimizing ionic transport in argyrodites: a unified view on the role of sulfur/halide distribution and local environments
Source: J Mater Chem A Mater. 2024 Sep 9;12(39):26596–611. doi: 10.1039/d4ta04628e (PMC11403572; doi:10.1039/d4ta04628e)
Supplement: TA-012-D4TA04628E-s001 [file TA-012-D4TA04628E-s001.pdf]

Supplementary Information (SI) for Journal of Materials Chemistry A.
This journal is © The Royal Society of Chemistry 2024

Optimizing Ionic Transport in Argyrodites: A Unified View on the Role of Sulfur/Halide Distribution and Local Environments

Anastasia K. Lavrinenko[a], Theodosios Famprikis[a], James A. Quirk[b], Victor Landgraf[a], Pedro B. Groszewicz[a,c], Jouke R. Heringa[a], Stef Smeets[d], Victor Azizi[d], Simone Ciarella[d], James A. Dawson[b], Marnix Wagemaker[a,*], Alexandros Vasileiadis[a,*]

[a] *Storage of Electrochemical Energy, Department of Radiation Science and Technology, Faculty of Applied Sciences, Delft University of Technology, Mekelweg 15, 2629JB, Delft, The Netherlands*

[b] *Chemistry – School of Natural and Environmental Sciences, Newcastle University, Newcastle upon Tyne NE1 7RU, UK*

[c] *Helmholtz Zentrum Berlin für Materialien und Energie, Hahn-Meitner-Platz 1, 14109, Berlin, Germany*

[d] *Netherlands eScience Center, Science Park 402, 1098 XH Amsterdam, The Netherlands*

*Corresponding authors: m.wagemaker@tudelft.nl, a.vasileiadis@tudelft.nl

Supplementary Information A - Structural Analysis

This section will introduce the structural framework of argyrodites, identifying and analyzing geometrical features induced by site disorder. This analysis will be compared with experimentally observed structural trends, demonstrating the relevance of our findings and their accurate representation of real systems.

We tested several configurations for structures experiencing site disorder in the anion sublattice. **Figure S1** demonstrates the total energies of the variations relative to the lowest energy. All energies are within 25 meV/atom, suggesting that all variations are probable, and the real material might exhibit a mix or an average of these configurations.

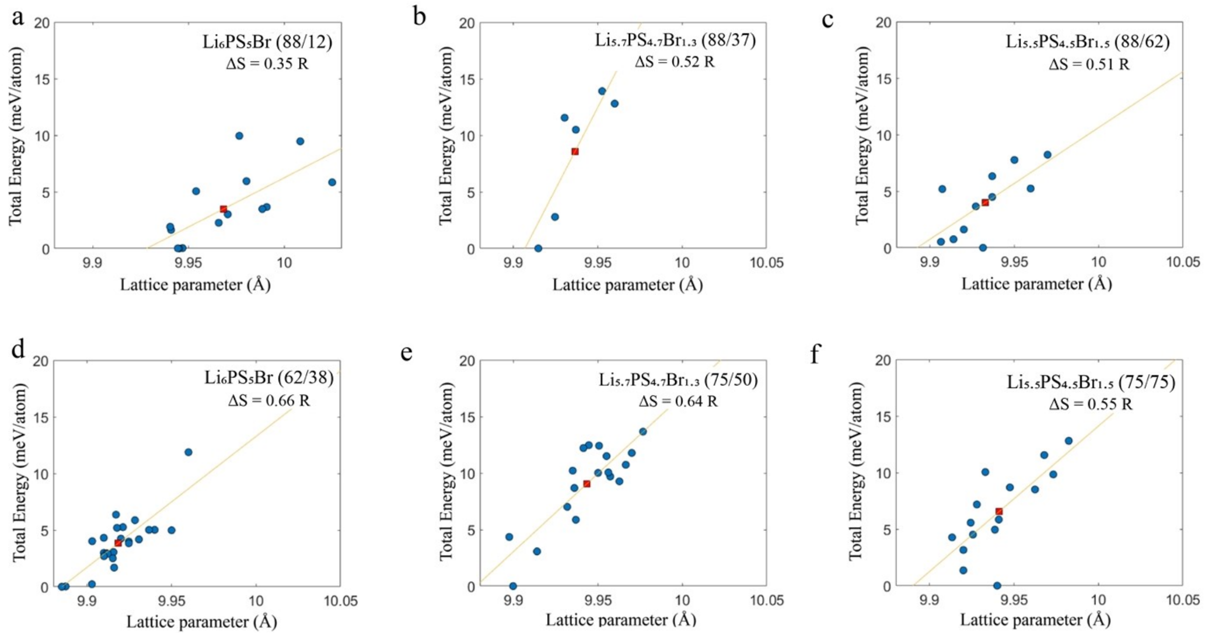

Figure S1. Energy vs. lattice parameter for the unique configurations for the six structures. The energies are referenced to the lowest energy per structure; the red point represents the average value.

Figure S2 demonstrates the occupation of lithium during 150 ps AIMD at 300 K. Several configurations were randomly chosen for each structure to allow for comparison. All configurations within one structure exhibit similar lithium distribution across the considered positions (T5, T2, T4), suggesting lithium distributions within specific configuration are representative of the overall behavior of the structure. All further analyses were performed on the lowest energy configuration of each structure to ensure accuracy and reliability in the results.

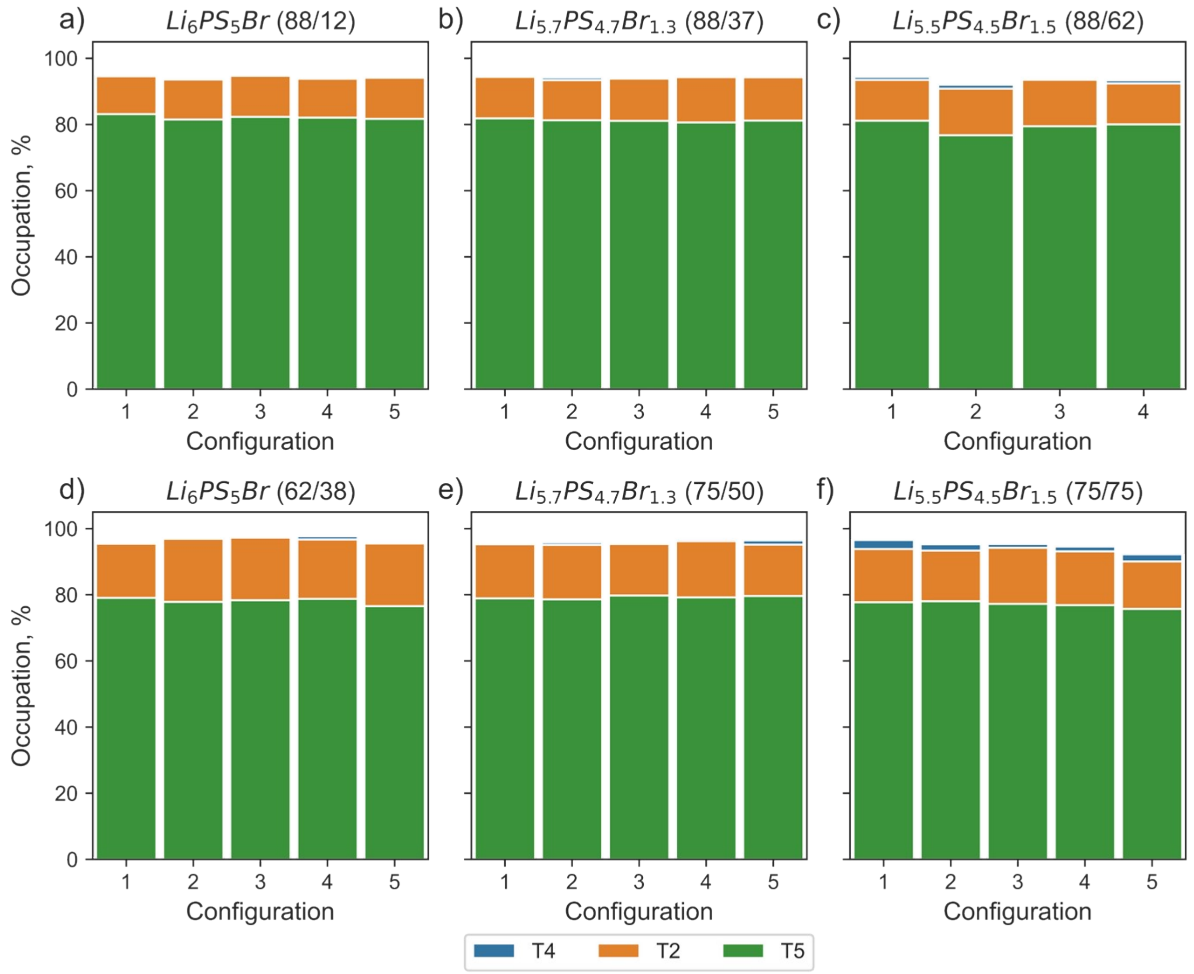

Figure S2. Comparison of lithium occupations within randomly selected configurations for each structure. Occupation analysis was performed using AIMD at 300 K.

The substitution of sulfur ($r_{S^{2-}}$ = 184 pm) with the larger bromine anion (r_{Br^-} = 196 pm) within a sublattice induces variation in steric and Coulombic interatomic interactions. This results in the redistribution of lithium, altering the lattice parameters accordingly. Halogen distribution in such a way results in a reduction in the lattice parameter with increasing configurational entropy. For structures with increased halogen content, introducing vacancies by substituting sulfur with bromine can further contribute to reducing the lattice parameter. The calculated lattice parameters are presented in **Figure S3a**, closely resembling the experimentally observed trend. Both ordered structures exhibit similar lattice parameters within 10.2-10.3 Å, and the calculated lattice parameters of the six structures representing experimental 4a/4d sublattice distributions agree within 1% with experimental values.

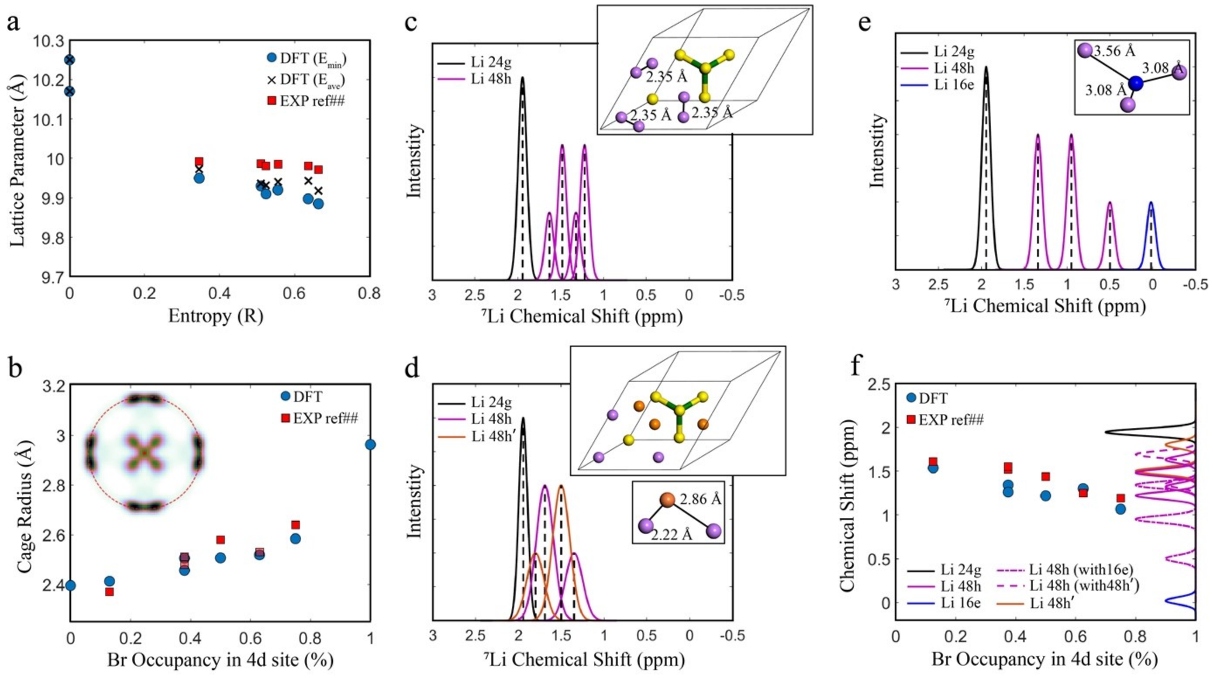

Figure S3. Structural characterization of argyrodites. **a)** Lattice parameters of relaxed structures compared to those measured experimentally[1]. The blue sphere indicates the lattice parameter of the lowest energy configuration of one structure. The x mark indicates the average lattice parameter of a structure. **b)** Cage radius surrounding the Wyckoff 4d site compared to experimental values from ref. [1]. The cage radius was calculated based on MD simulation at 300 K on the lowest energy configuration. **c)** Computed NMR peaks when all six Li occupy 48h positions (purple lines). The peak when all six Li occupies 24g positions is plotted for reference (black line). **d)** NMR spectrum when three Li occupy 48h positions (purple lines) and three Li occupy 48h' positions (orange lines). The peak when all six Li occupies 24g positions is plotted for reference (black line). **e)** NMR peaks when five Li occupy 48h positions (purple lines) and one Li occupies 16e positions (blue line). The peak when all six Li occupies 24g positions is plotted for reference (black line). **f)** Calculated (blue circles) and experimental NMR peaks (red circles) vs. occupancy in the 4d site. NMR peaks were calculated by averaging the DFT-determined signal weighted per environment and the experimentally determined Li occupancies[1].

The redistribution of Li^+ is further supported by the change in cage radius (**Figure S3b**) surrounding the Wyckoff 4d. The radius was determined as the average distance between the 4d site and lithium positions during each step of AIMD simulations at 300 K, with an example projection of the lithium density around the 4d site illustrated as an inlet in **Figure S3b**. The calculated radii align closely with experimentally determined values[1].

We further performed calculations of Nuclear Magnetic Resonance (NMR) spectroscopy parameters to gain deeper insight into the redistribution of lithium ions within the argyrodite structure. NMR parameters offer a direct pathway to compare observables from the computational structural models to experimental data, here with particular attention to ^7Li NMR and how the ^7Li chemical shift is influenced by anion disorder. Our computational approach recreated environments with varying Li occupations in primitive cells, adhering to stoichiometry and maintaining the minimum Li-Li distances allowed[2]. Based on these criteria, we modeled four distinct Li configurations: Li exclusively at 24g (T5a) positions, Li exclusively at 48h (T5) positions, Li in a combination of T5 and T2 positions, and Li in a mix of T4 and T5 positions. The chemical shifts of Li representing the latter three scenarios are shown in

Figures S3c, **d**, and **e**, with the peak from the first scenario plotted as a reference on top for comparison.

When studying argyrodites experimentally, a single ^{7}Li NMR peak is observed. In the fast regime, this peak results from the dynamic exchange among various chemical environments experienced by lithium in the sample and its position reflects an average of the chemical shift of these environments[3]. Accordingly, we weighted the position of calculated signals of each environment based on their intensity (number of Li in each environment) and their experimentally determined Li occupancies for each structure. The results are displayed in **Figure S3f**. As lithium occupancies vary due to different anionic disorder, so do Li's local environments. For instance, when Li occupies the T4 site, the Li-Li/S/Br distances result in a ^{7}Li NMR signal at 0 ppm for the T4 site (**Figure S3e**). Meanwhile, the peaks for Li ions remaining at the 48h (T5) positions shift to lower ppm values (**Figure S3e**) if in the vicinity of occupied T4 sites, compared to higher chemical shift values for Li ions when residing solely on 48h (T5) positions (**Figure S3c**). Consequently, structures with high Li occupancy at the T4 site typically exhibit NMR peak centred at lower ppm values compared to those with more Li occupancy at the T5a position. This trend is observed in the computed average values for ^{7}Li chemical shift (**Figure S3f**) and demonstrates how Li redistribution affects chemical shifts amid varying anionic disorder.

Experimental results have shown a similar trend for ^{7}Li chemical shift values: the Li peak shifts from +1.6 ppm in structures with less than 10% Br occupancy to +1.2 ppm when Br occupancy reaches 70% at the 4d site[1]. Furthermore, it advocates for the potential of ^{7}Li (or ^{6}Li) NMR as an experimental means to gauge the extent of anion mixing in these materials.

To translate the calculated values into chemical shifts comparable with experiments, we used the linear response method with reference structures Li_2S, Li_2O, Li_2CO_3, LiF LiCl, and LiBr, as depicted in Figure S4.

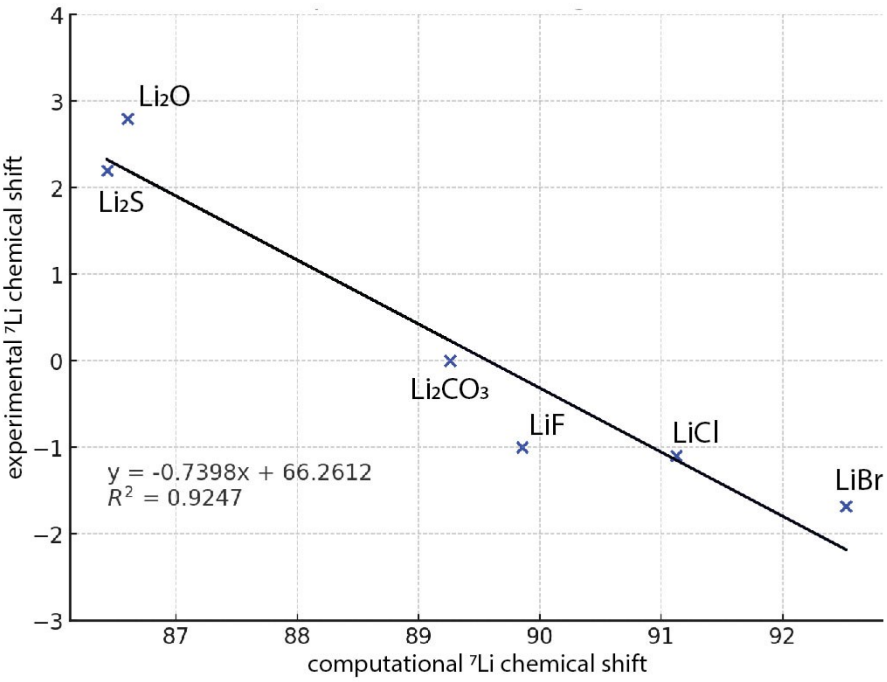

Figure S4. Linear response between experimentally and computationally determined ^{7}Li chemical shifts. References for the experimental values are reported in Table S1.

Supplementary Information B - Diffusion Properties

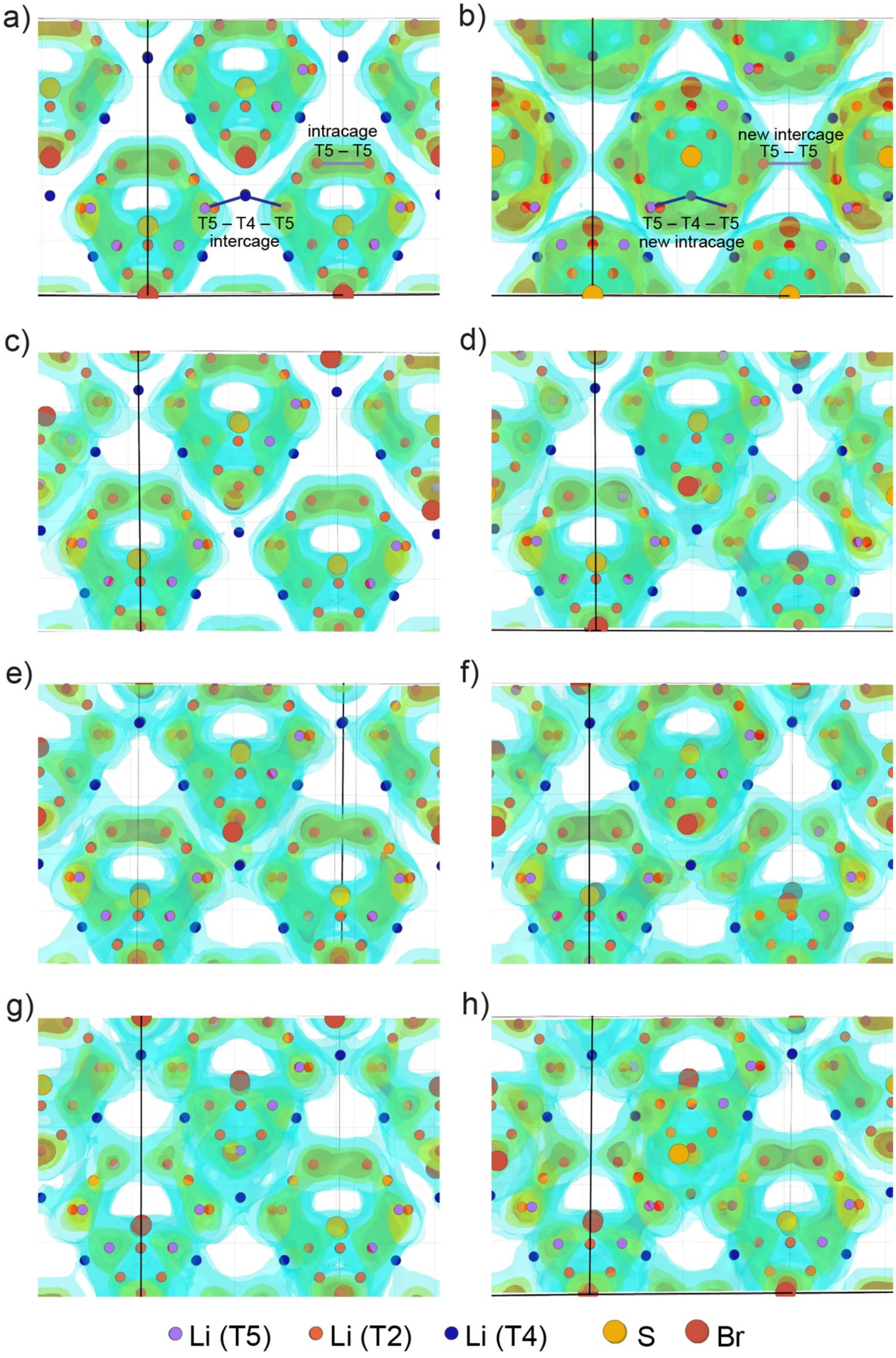

Figure S5. The probability density of Li^+ at different tetrahedral sites: T5 (violet), T2 (orange), and T4 (blue), derived from AIMD simulations at 650K. Additionally, the positions of bromine (red) and sulfur (yellow) are highlighted. **a)** Li_6PS_5Br, ΔS = 0.00 R; **b)** Li_6PS_5Br, ΔS = 0.00* R; **c)** Li_6PS_5Br, ΔS = 0.35 R; **d)** Li_6PS_5Br, ΔS = 0.66 R; **e)** $Li_{5.7}PS_{4.7}Br_{1.3}$, ΔS = 0.52 R; **f)** $Li_{5.7}PS_{4.7}Br_{1.3}$, ΔS = 0.64 R; **g)** $Li_{5.5}PS_{4.5}Br_{1.5}$, ΔS = 0.51 R; **h)** $Li_{5.5}PS_{4.5}Br_{1.5}$, ΔS = 0.55 R.

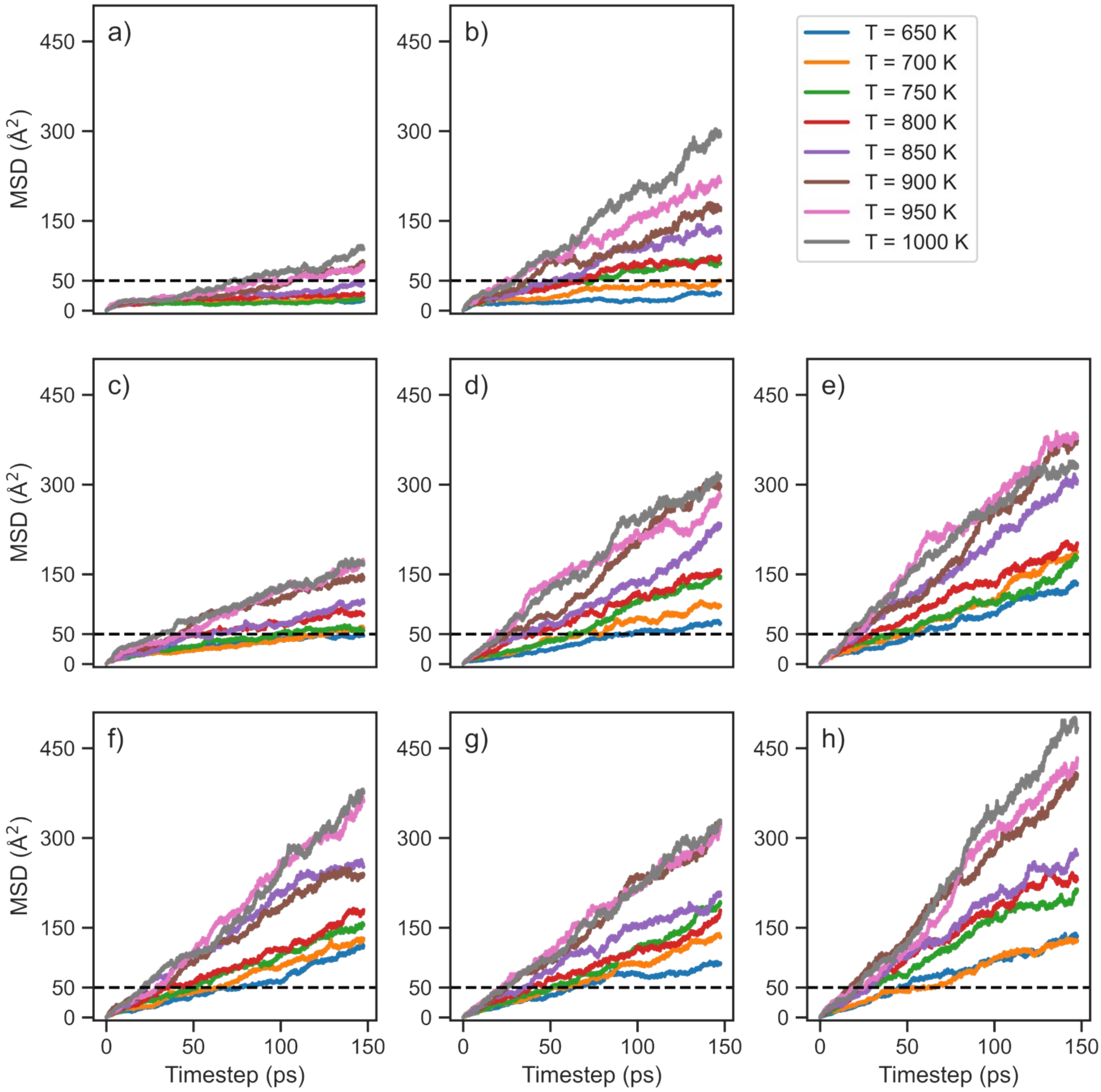

Figure S6. Mean squared displacements obtained from AIMD simulations performed at 650-1000K: **a)** Li_6PS_5Br, ΔS = 0.00 R; **b)** Li_6PS_5Br, ΔS = 0.00* R; **c)** Li_6PS_5Br, ΔS = 0.35 R; **d)** $Li_{5.7}PS_{4.7}Br_{1.3}$, ΔS = 0.52 R; **e)** $Li_{5.5}PS_{4.5}Br_{1.5}$, ΔS = 0.51 R; **f)** Li_6PS_5Br, ΔS = 0.66 R; **g)** $Li_{5.7}PS_{4.7}Br_{1.3}$, ΔS = 0.64 R; **h)** $Li_{5.5}PS_{4.5}Br_{1.5}$, ΔS = 0.55 R.

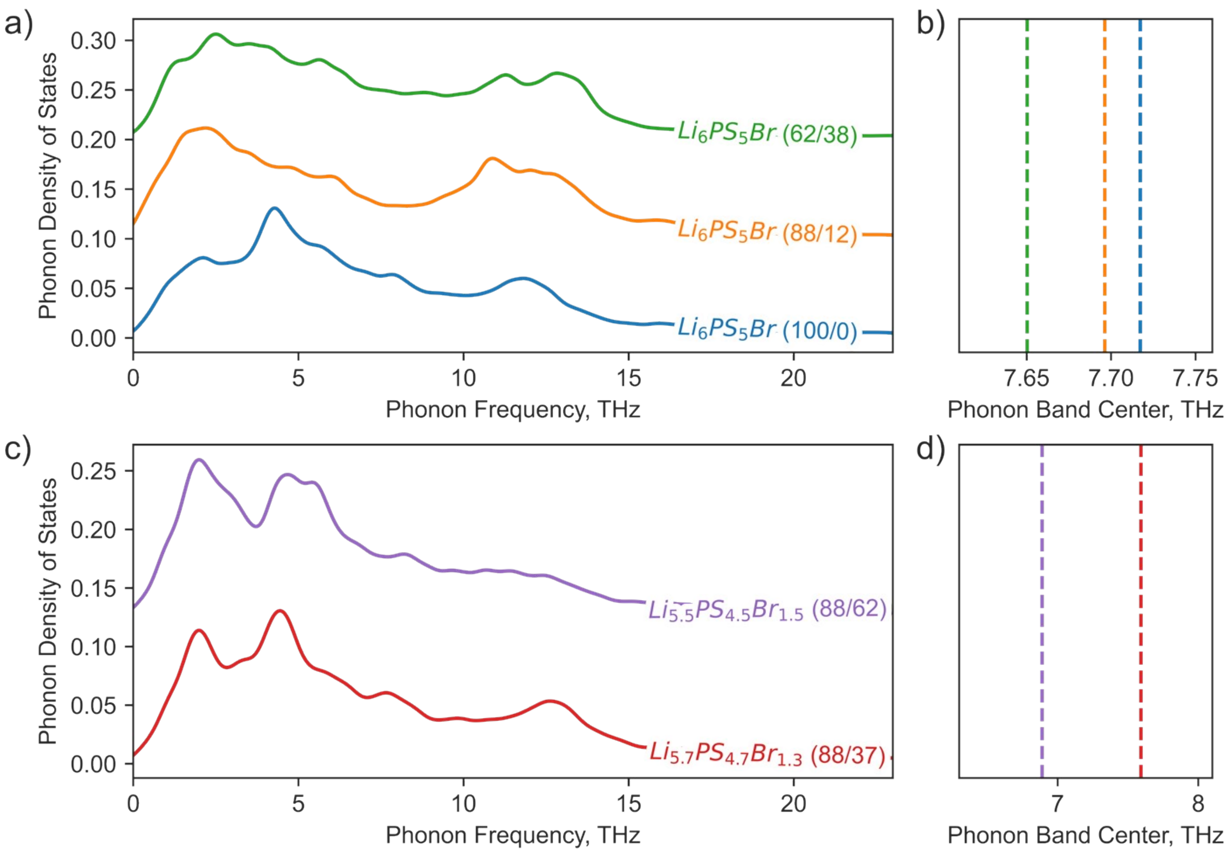

Figure S7. The phonon density of states (DOS) calculated from AIMD at 300 K, alongside the band centre of a projected phonon DOS. The decrease in the band centre with an increase in both configurational entropy (b) and bromine content (d) indicates a softening of the lattice.

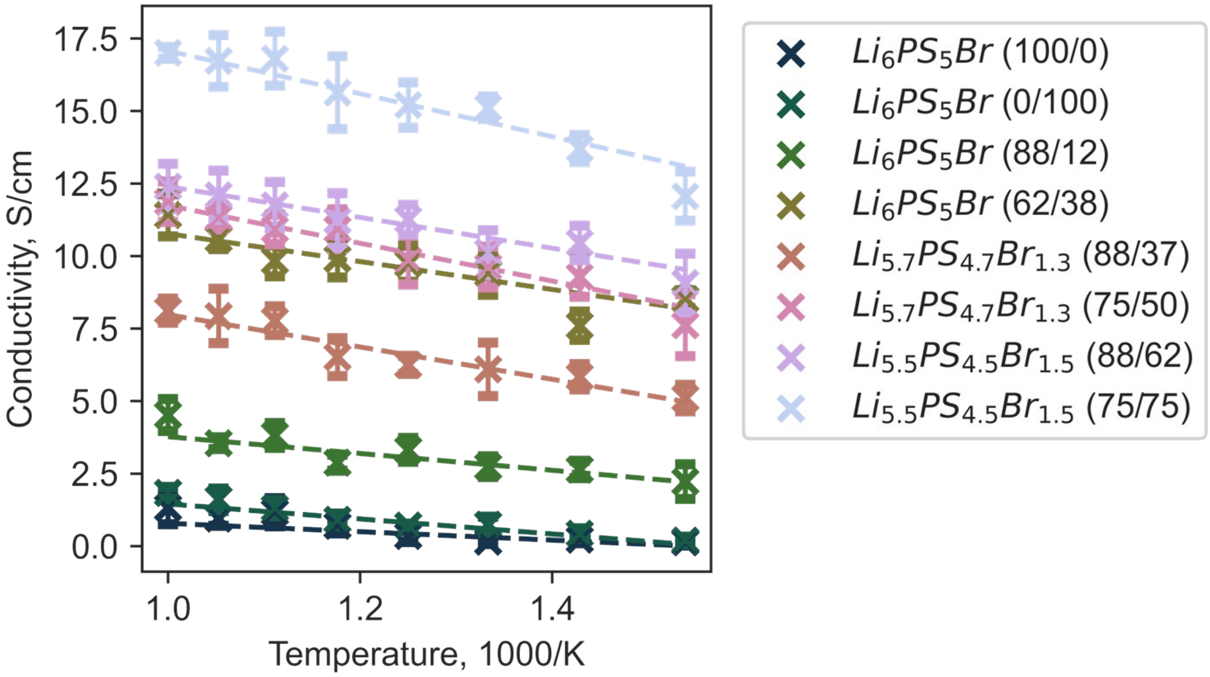

Figure S8. Arrhenius plot based on conductivities calculated from rate-limiting jumps in the temperature range of 650-1000 K.

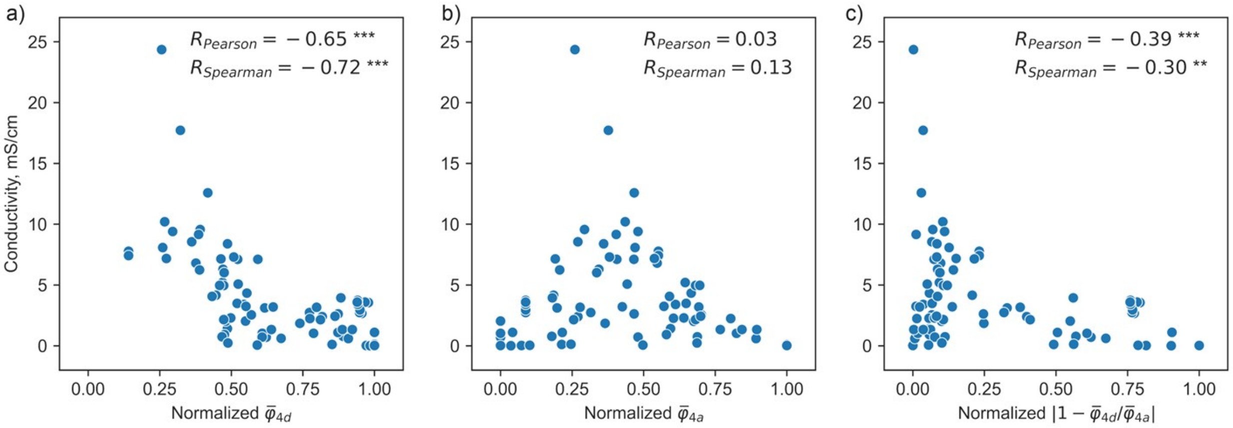

Figure S9. Correlation between conductivity and individual descriptors. **a)** Relationship between the normalized average ionic potential of the 4d sublattice ($\bar{\varphi}_{4d}$) and the corresponding conductivity values. **b)** Relationship between the normalized average ionic potential of the 4a sublattice ($\bar{\varphi}_{4a}$) and the corresponding conductivity values. **c)** Correlation of the normalized absolute deviation of the average ionic potentials ratio from one $\left(\left|1 - \frac{\bar{\varphi}_{4d}}{\bar{\varphi}_{4a}}\right|\right)$ with conductivity. Pearson's ($R_{Pearson}$) and Spearman's rank ($R_{Spearman}$) correlation coefficients are calculated between conductivity and the corresponding descriptors, with significance levels indicated ($p < 0.01$ as ** and $p < 0.001$ as ***).

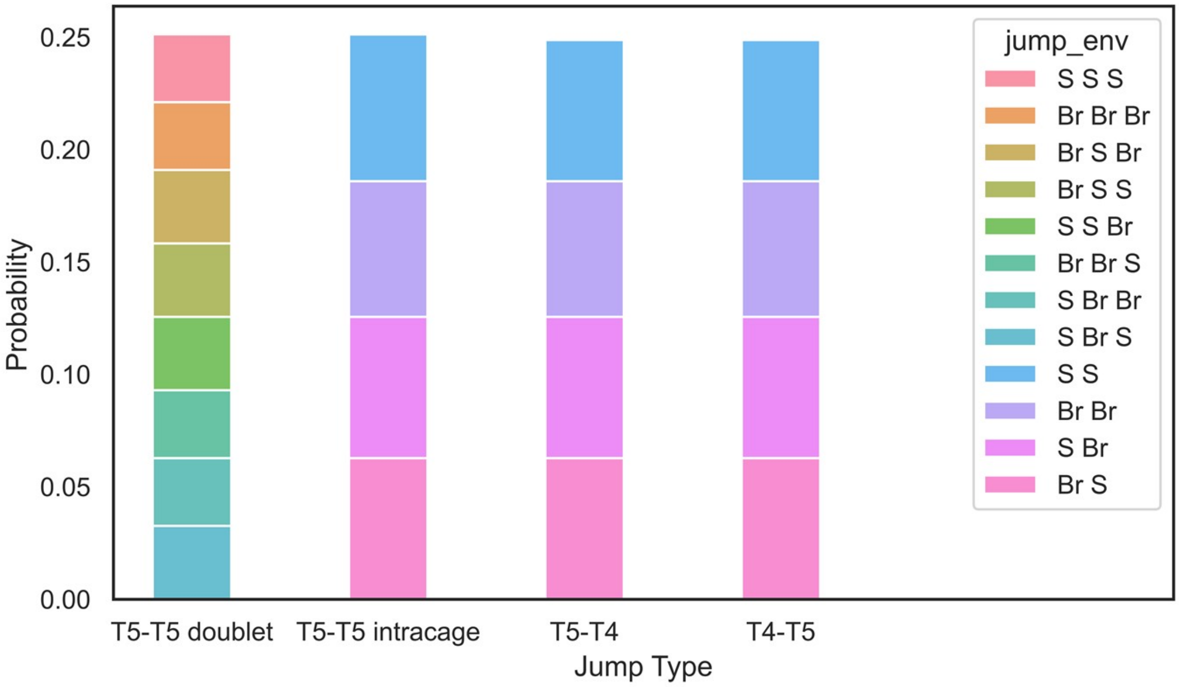

Figure S10. Probability of local environment appearance per jump type in the composition Li_6PS_5Br (50/50). The occurrence of each environment was calculated based on a 5x5x5 supercell. Fifty

configurations were generated using the pymatgen[4] library with random distribution of sulfur and bromine across the 4a and 4d sublattices.

Table S1. Experimentally determined ^{7}Li NMR chemical shifts.

**Structure**	**Chemical Shift**	**Reference**
LiBr	-1.68	[5]
LiCl	-1.1	[5]
LiF	-1	[5]
Li_2O	2.8	[5]
Li_2CO_3	0	[5]
Li_2S	2.2	[6]

Table S2. The activation energies associated with different lithium ion jump types and environments within Li_6PS_5Br structures, showcasing variations across samples with different degrees of site disorder and ordering. These values are calculated from AIMD simulations conducted at 650 K.

Jump Type	Jump Environment			Li_6PS_5Br (100/0)	Li_6PS_5Br (88/12)		Li_6PS_5Br (62/38)				Li_6PS_5Br (0/100)	Average	Standard Deviation
	4a	4d	4a	(1)	(1)	(2)	(1)	(2)	(3)	(4)	(1)		
T5-T5 (doublet)	Br	S	Br	0.134	0.141	0.139	0.139	0.143	0.137	0.140		0.139	0.003
	Br	S	S		0.167	0.174	0.172	0.174	0.185	0.180		0.176	0.006
	S	S	Br		0.195	0.196	0.192	0.191	0.200	0.196		0.195	0.003
	Br	Br	Br		0.219	0.218	0.222	0.238	0.229	0.241		0.228	0.009
	S	S	S				0.244	0.247	0.223	0.244		0.239	0.010
	Br	Br	S		0.345		0.263	0.265	0.267	0.255		0.279	0.033
	S	Br	Br		0.314		0.291	0.301	0.307	0.298		0.302	0.008
	S	Br	S				0.320	0.331	0.307	0.345	0.353	0.331	0.017
T5-T5 (intracage)	S	Br	S		0.169		0.179	0.178	0.187	0.195	0.164	0.178	0.01
	Br	Br	Br		0.179	0.184	0.183	0.178	0.188	0.182		0.182	0.003
	S	S	S		0.231	0.211	0.219	0.216	0.213	0.209		0.217	0.007
	Br	S	Br	0.219	0.22	0.226	0.212	0.219	0.216	0.211		0.218	0.005
T5-T4	S	Br			0.153		0.155	0.160	0.157	0.148	0.141	0.152	0.006
	Br	Br			0.267	0.243	0.224	0.232	0.233	0.225		0.237	0.015
	S	S			0.268	0.271	0.242	0.247	0.249	0.274		0.258	0.013
	Br	S		0.347	0.331	0.332	0.319	0.303	0.316	0.314		0.323	0.013
T4-T5	Br	Br			0.036	0.032	0.021	0.02	0.029	0.031		0.028	0.006
	S	Br			0.049		0.049	0.045	0.045	0.036	0.023	0.041	0.009
	Br	S		-0.021	0.031	0.052	0.081	0.068	0.086	0.081		0.054	0.036
	S	S			0.073	0.018	0.094	0.097	0.087	0.104		0.079	0.029

Table S3. Experimental data, including measured conductivities and elemental occupation at the 4d and 4a positions, gathered from literature for argyrodite structures and their modifications. Additionally, the calculated average ionic potentials for the 4d ($\bar{\varphi}_{4d}$) and 4a ($\bar{\varphi}_{4a}$) sublattices are presented.

Structure	Conductivity, mS/cm	Occupation (4d)	Occupation (4a)	Reference	$\bar{\varphi}_{4d}$	$\bar{\varphi}_{4a}$
$Li_{5.5}PS_{4.5}Br_{1.5}$	8.550	$Br_{0.70}$ $S_{0.30}$	$Br_{0.80}$ $S_{0.20}$	[1]	0.683	0.626
$Li_{5.7}PS_{4.7}Br_{1.3}$	6.290	$Br_{0.58}$ $S_{0.42}$	$Br_{0.72}$ $S_{0.28}$	[1]	0.752	0.672
$Li_{5.5}PS_{4.5}Br_{1.5}$	6.240	$Br_{0.67}$ $S_{0.33}$	$Br_{0.87}$ $S_{0.13}$	[1]	0.701	0.585
Li_6PS_5Br	3.210	$Br_{0.39}$ $S_{0.61}$	$Br_{0.63}$ $S_{0.37}$	[1]	0.862	0.724
$Li_{5.7}PS_{4.7}Br_{1.3}$	3.114	$Br_{0.42}$ $S_{0.58}$	$Br_{0.88}$ $S_{0.12}$	[1]	0.845	0.579
Li_6PS_5Br	0.777	$Br_{0.12}$ $S_{0.88}$	$Br_{0.90}$ $S_{0.10}$	[1]	1.018	0.568
$Li_{5.6}PS_{4.6}I_{1.4}$	2.040	$S_{0.555}$ $I_{0.455}$	I	[7]	0.802	0.455
$Li_{5.7}PS_{4.7}I_{1.3}$	1.029	$S_{0.608}$ $I_{0.392}$	I	[7]	0.839	0.455
$Li_{5.8}PS_{4.8}I_{1.2}$	0.709	$S_{0.622}$ $I_{0.378}$	I	[7]	0.848	0.455
$Li_{5.9}PS_{4.9}I_{1.1}$	0.615	$S_{0.674}$ $I_{0.326}$	I	[7]	0.881	0.455
$Li_{5.5}PS_{4.5}Cl_{0.89}Br_{0.61}$	7.780	$Cl_{0.791}$ $Br_{0.209}$	$Cl_{0.098}$ $Br_{0.401}$ $S_{0.501}$	[8]	0.544	0.803
$Li_{5.5}PS_{4.41}O_{0.09}Cl_{0.89}Br_{0.61}$	7.420	$Cl_{0.791}$ $Br_{0.209}$	$Cl_{0.098}$ $Br_{0.401}$ $S_{0.501}$	[8]	0.544	0.803
$Li_{5.5}PS_{4.5}Cl_{1.5}$	9.400	$S_{0.166}$ $Cl_{0.834}$	$S_{0.385}$ $Cl_{0.615}$	[9]	0.641	0.758
$Li_{5.5}PS_{4.5}Cl_{0.8}Br_{0.7}$	9.560	$Cl_{0.555}$ $S_{0.289}$ $Br_{0.147}$	$Cl_{0.253}$ $S_{0.21}$ $Br_{0.553}$	[10]	0.702	0.640
$Li_{5.5}PS_{4.5}Cl_{1.2}Br_{0.3}$	8.080	$Cl_{0.716}$ $S_{0.136}$ $Br_{0.145}$	$Cl_{0.49}$ $S_{0.403}$ $Br_{0.155}$	[10]	0.619	0.752
$Li_{5.5}PS_{4.5}Cl_{0.4}Br_{1.1}$	7.150	$Cl_{0.341}$ $S_{0.39}$ $Br_{0.277}$	$Cl_{0.059}$ $S_{0.108}$ $Br_{0.823}$	[10]	0.748	0.576
$Li_{5.5}PS_{4.5}Cl_{1.5}$	7.110	$Cl_{0.903}$ $S_{0.97}$	$Cl_{0.632}$ $S_{0.368}$	[10]	0.829	0.749
$Li_{5.5}PS_{4.5}Br_{1.5}$	4.160	$Br_{0.607}$ $S_{0.393}$	$Br_{0.893}$ $S_{0.107}$	[10]	0.737	0.572
Li_6PS_5Cl	0.740	$S_{0.37}$ $Cl_{0.63}$	$S_{0.63}$ $Cl_{0.37}$	[11]	0.750	0.889
Li_6PS_5Br	0.720	$S_{0.57}$ $Br_{0.43}$	$S_{0.43}$ $Br_{0.57}$	[11]	0.839	0.758
Li_6PS_5I	0.000	S	I	[11]	1.087	0.455
$Li_{5.4}Al_{0.2}PS_5Br$	2.400	$S_{0.80}$ $Br_{0.20}$	$S_{0.20}$ $Br_{0.80}$	[12]	0.972	0.626
Li_7PS_6	0.110	S	S	[13]	1.087	1.087
Li_6PS_5I	0.029	S	I	[13]	1.087	0.455
$Li_{5.6}PS_{4.6}ClBr_{0.4}$	9.160	$S_{0.29}$ $Cl_{0.5}$ $Br_{0.21}$	$S_{0.31}$ $Cl_{0.5}$ $Br_{0.19}$	[14]	0.699	0.710
Li_6PS_5Cl	4.960	$S_{0.377}$ $Cl_{0.623}$	$S_{0.623}$ $Cl_{0.377}$	[14]	0.754	0.885
$Li_{6.3}P_{0.9}Cu_{0.1}S_{4.9}Cl_{1.1}$	4.328	$S_{0.4727}$ $Cl_{0.5273}$	$S_{0.6045}$ $Cl_{0.3955}$	[15]	0.805	0.876
$Li_{6.6}P_{0.8}Cu_{0.2}S_{4.8}Cl_{1.2}$	3.490	$S_{0.4325}$ $Cl_{0.5675}$	$S_{0.5839}$ $Cl_{0.4161}$	[15]	0.784	0.865

Li_6PS_5Cl	2.543	$S_{0.4911}$ $Cl_{0.5089}$	$S_{0.6472}$ $Cl_{0.3528}$	[15]	0.815	0.898
$Li_{6.9}P_{0.7}Cu_{0.3}S_{4.7}Cl_{1.3}$	2.267	$S_{0.4082}$ $Cl_{0.5918}$	$S_{0.5315}$ $Cl_{0.4685}$	[15]	0.771	0.837
$Li_{7.2}P_{0.6}Cu_{0.4}S_{4.6}Cl_{1.4}$	1.419	$S_{0.3923}$ $Cl_{0.6077}$	$S_{0.5192}$ $Cl_{0.4808}$	[15]	0.762	0.830
$Li_{7.5}P_{0.5}Cu_{0.5}S_{4.5}Cl_{1.5}$	0.923	$S_{0.3799}$ $Cl_{0.6201}$	$S_{0.5027}$ $Cl_{0.4973}$	[15]	0.756	0.821
Li_6PS_5Cl	3.400	$S_{0.46}$ $Cl_{0.54}$	$S_{0.54}$ $Cl_{0.46}$	[16]	0.798	0.841
$Li_{5.35}Ca_{0.1}PS_{4.5}Cl_{1.55}$	10.200	$S_{0.133}$ $Cl_{0.867}$	$S_{0.332}$ $Cl_{0.668}$	[17]	0.624	0.730
$Li_{5.55}Ca_{0.1}PS_{4.75}Cl_{1.25}$	6.800	$S_{0.262}$ $Cl_{0.738}$	$S_{0.464}$ $Cl_{0.536}$	[17]	0.693	0.800
$Li_{5.7}Ca_{0.15}PS_5Cl$	5.200	$S_{0.372}$ $Cl_{0.628}$	$S_{0.58}$ $Cl_{0.42}$	[17]	0.751	0.862
$Li_{5.3}PS_{4.3}ClBr_{0.7}$	24.354	$S_{0.15}$ $Cl_{0.5}$ $Br_{0.36}$	$S_{0.15}$ $Cl_{0.5}$ $Br_{0.34}$	[18]	0.617	0.619
$Li_{5.5}PS_{4.5}ClBr_{0.5}$	17.717	$S_{0.22}$ $Cl_{0.5}$ $Br_{0.28}$	$S_{0.28}$ $Cl_{0.5}$ $Br_{0.22}$	[18]	0.658	0.693
$Li_{5.7}PS_{4.7}ClBr_{0.3}$	12.584	$S_{0.32}$ $Cl_{0.57}$ $Br_{0.11}$	$S_{0.38}$ $Cl_{0.43}$ $Br_{0.18}$	[18]	0.719	0.750
Li_6PS_5Cl	4.973	$S_{0.36}$ $Cl_{0.64}$	$S_{0.64}$ $Cl_{0.36}$	[18]	0.745	0.895
Li_6PS_5CN	0.230	$S_{0.38}$ $(CN)_{0.62}$	$S_{0.62}$ $(CN)_{0.38}$	[19]	0.763	0.889
Li_6PS_5Br	0.060	$S_{0.551}$ $Br_{0.449}$	$S_{0.449}$ $Br_{0.551}$	[19]	0.828	0.769
$Li_{6.25}PS_{5.25}Cl_{0.75}$	3.190	$S_{0.581}$ $Cl_{0.419}$	$S_{0.636}$ $Cl_{0.364}$	[20]	0.863	0.892
$Li_{6.25}PS_{5.25}Br_{0.75}$	2.624	$S_{0.861}$ $Br_{0.139}$	$S_{0.415}$ $Br_{0.585}$	[20]	1.007	0.750
$Li_{6.5}PS_{5.5}Br_{0.5}$	2.430	$S_{0.849}$ $Br_{0.151}$	$S_{0.670}$ $Br_{0.330}$	[20]	1.000	0.897
$Li_{6.5}PS_{5.5}Cl_{0.5}$	2.241	$S_{0.733}$ $Cl_{0.267}$	$S_{0.768}$ $Cl_{0.232}$	[20]	0.944	0.963
Li_6PS_5Br	2.151	$S_{0.793}$ $Br_{0.207}$	$S_{0.184}$ $Br_{0.816}$	[20]	0.968	0.616
$Li_{6.75}PS_{5.75}Cl_{0.25}$	1.336	$S_{0.867}$ $Cl_{0.133}$	$S_{0.876}$ $Cl_{0.124}$	[20]	1.016	1.021
$Li_{6.75}PS_{5.75}Br_{0.25}$	1.333	$S_{0.915}$ $Br_{0.085}$	$S_{0.828}$ $Br_{0.172}$	[20]	1.038	0.988
$Li_{6.4}PS_{5.4}I_{0.6}$	0.128	S	$S_{0.246}$ $I_{0.754}$	[20]	1.087	0.610
$Li_{6.2}PS_{5.2}I_{0.8}$	0.039	S	$S_{0.102}$ $I_{0.898}$	[20]	1.087	0.519
Li_6PS_5I	0.003	S	I	[20]	1.087	0.455
$Li_{5.85}PSe_{4.85}Br_{1.15}$	8.389	$Se_{0.508}$ $Br_{0.499}$	$Se_{0.342}$ $Br_{0.651}$	[21]	0.762	0.682
$Li_{5.9}PSe_{4.9}Br_{1.1}$	7.299	$Se_{0.525}$ $Br_{0.462}$	$Se_{0.375}$ $Br_{0.638}$	[21]	0.776	0.695
$Li_{5.95}PSe_{4.95}Br_{1.05}$	7.109	$Se_{0.543}$ $Br_{0.446}$	$Se_{0.407}$ $Br_{0.604}$	[21]	0.785	0.711
$Li_{5.8}PSe_{4.8}Br_{1.2}$	6.019	$Se_{0.481}$ $Br_{0.502}$	$Se_{0.319}$ $Br_{0.698}$	[21]	0.755	0.667
Li_6PSe_5Br	5.071	$Se_{0.550}$ $Br_{0.447}$	$Se_{0.450}$ $Br_{0.553}$	[21]	0.786	0.734

$Li_6PS_{4.8}Se_{0.2}Br$	3.947	$S_{0.697}$ $Se_{0.2}$ $Br_{0.102}$	$S_{0.102}$ $Br_{0.897}$	[22]	1.013	0.569
$Li_6PS_{4.1}Se_{0.9}Br$	3.733	$S_{0.515}$ $Se_{0.485}$	Br	[22]	1.050	0.510
Li_6PS_4SeBr	3.591	$S_{0.504}$ $Se_{0.496}$	Br	[22]	1.049	0.510
$Li_6PS_{4.6}Se_{0.4}Br$	3.591	$S_{0.729}$ $Se_{0.271}$	Br	[22]	1.066	0.510
$Li_6PS_{4.7}Se_{0.3}Br$	3.556	$S_{0.825}$ $Se_{0.175}$	Br	[22]	1.074	0.510
$Li_6PS_{4.2}Se_{0.8}Br$	3.413	$S_{0.518}$ $Se_{0.482}$	Br	[22]	1.050	0.510
$Li_6PS_{4.9}Se_{0.1}Br$	3.164	$S_{0.691}$ $Se_{0.1}$ $Br_{0.208}$	$S_{0.208}$ $Br_{0.791}$	[22]	0.959	0.630
$Li_6PS_{4.3}Se_{0.7}Br$	2.951	$S_{0.582}$ $Se_{0.418}$	Br	[22]	1.055	0.510
$Li_6PS_{4.4}Se_{0.6}Br$	2.738	$S_{0.568}$ $Se_{0.432}$	Br	[22]	1.054	0.510
Li_6PS_5Br	2.738	$S_{0.751}$ $Br_{0.249}$	$S_{0.249}$ $Br_{0.751}$	[22]	0.943	0.654
$Li_6PS_{4.5}Se_{0.5}Br$	2.667	$S_{0.637}$ $Se_{0.363}$	Br	[22]	1.059	0.510
$Li_{5.5}PS_{4.5}Cl_{1.5}$	7.177	$S_{0.140}$ $Cl_{0.860}$	$S_{0.453}$ $Cl_{0.547}$	[23]	0.627	0.795
$Li_{5.75}PS_{4.75}Cl_{1.25}$	4.064	$S_{0.329}$ $Cl_{0.671}$	$S_{0.515}$ $Cl_{0.485}$	[23]	0.728	0.828
Li_6PS_5Cl	2.161	$S_{0.377}$ $Cl_{0.623}$	$S_{0.623}$ $Cl_{0.377}$	[23]	0.754	0.885
$Li_{6.25}PS_{5.25}Cl_{0.75}$	1.338	$S_{0.574}$ $Cl_{0.426}$	$S_{0.725}$ $Cl_{0.275}$	[23]	0.859	0.940
$Li_{6.5}PS_{5.5}Cl_{0.5}$	1.029	$S_{0.748}$ $Cl_{0.252}$	$S_{0.792}$ $Cl_{0.208}$	[23]	0.952	0.976
$Li_{6.75}PS_{5.75}Cl_{0.25}$	0.592	$S_{0.892}$ $Cl_{0.108}$	$S_{0.873}$ $Cl_{0.127}$	[23]	1.029	1.019
Li_7PS_6	0.026	S	S	[23]	1.087	1.087
$Li_{6.55}P_{0.45}Si_{0.55}S_5I$	1.100	S	$S_{0.042}$ $I_{0.958}$	[24]	1.087	0.481
$Li_6PS_5Cl_{0.5}Br_{0.5}$	3.243	$S_{0.492}$ $Cl_{0.211}$ $Br_{0.297}$	$S_{0.508}$ $Cl_{0.289}$ $Br_{0.203}$	[25]	0.803	0.815
$Li_6PS_5Cl_{0.75}Br_{0.25}$	2.296	$S_{0.418}$ $Cl_{0.435}$ $Br_{0.148}$	$S_{0.582}$ $Cl_{0.315}$ $Br_{0.102}$	[25]	0.769	0.860
Li_6PS_5Cl	2.018	$S_{0.385}$ $Cl_{0.615}$	$S_{0.615}$ $Cl_{0.385}$	[25]	0.758	0.881
$Li_6PS_5Cl_{0.25}Br_{0.75}$	1.849	$S_{0.704}$ $Cl_{0.140}$ $Br_{0.156}$	$S_{0.296}$ $Cl_{0.110}$ $Br_{0.594}$	[25]	0.922	0.686
Li_6PS_5Br	1.099	$S_{0.86}$ $Br_{0.14}$	$S_{0.14}$ $Br_{0.86}$	[25]	1.006	0.591
$Li_6PS_5Br_{0.75}I_{0.25}$	0.116	$S_{0.845}$ $Br_{0.087}$ $I_{0.069}$	$S_{0.155}$ $Br_{0.663}$ $I_{0.181}$	[25]	0.993	0.590
$Li_6PS_5Br_{0.5}I_{0.5}$	0.025	$S_{0.97}$ $Br_{0.01}$ $I_{0.02}$	$S_{0.03}$ $Br_{0.49}$ $I_{0.48}$	[25]	1.069	0.501
$Li_6PS_5Br_{0.25}I_{0.75}$	0.005	$S_{0.985}$ $Br_{0.007}$ $I_{0.009}$	$S_{0.015}$ $Br_{0.243}$ $I_{0.741}$	[25]	1.077	0.478
Li_6PS_5I	0.001	S	I	[25]	1.087	0.455
Li_6SbS_5I	0.028	S	I	[26]	1.087	0.455

References

(1) Gautam, A.; Al-Kutubi, H.; Famprikis, T.; Ganapathy, S.; Wagemaker, M. Exploring the Relationship Between Halide Substitution, Structural Disorder, and Lithium Distribution in Lithium Argyrodites ($Li_{6-x}PS_{5-x}Br_{1+x}$). *Chemistry of Materials* **2023**, *35* (19), 8081–8091. https://doi.org/10.1021/acs.chemmater.3c01525.

(2) Wang, P.; Liu, H.; Patel, S.; Feng, X.; Chien, P.-H.; Wang, Y.; Hu, Y.-Y. Fast Ion Conduction and Its Origin in Li6–XPS5–XBr1+x. *Chemistry of Materials* **2020**, *32* (9), 3833–3840. https://doi.org/10.1021/acs.chemmater.9b05331.

(3) Bain, A. D. Chemical Exchange; 2008; pp 23–48. https://doi.org/10.1016/S0066-4103(07)63002-6.

(4) Ong, S. P.; Richards, W. D.; Jain, A.; Hautier, G.; Kocher, M.; Cholia, S.; Gunter, D.; Chevrier, V. L.; Persson, K. A.; Ceder, G. Python Materials Genomics (Pymatgen): A Robust, Open-Source Python Library for Materials Analysis. *Comput Mater Sci* **2013**, *68*, 314–319. https://doi.org/10.1016/j.commatsci.2012.10.028.

(5) Meyer, B. M.; Leifer, N.; Sakamoto, S.; Greenbaum, S. G.; Grey, C. P. High Field Multinuclear NMR Investigation of the SEI Layer in Lithium Rechargeable Batteries. *Electrochemical and Solid-State Letters* **2005**, *8* (3), A145. https://doi.org/10.1149/1.1854117.

(6) Szczuka, C.; Karasulu, B.; Groh, M. F.; Sayed, F. N.; Sherman, T. J.; Bocarsly, J. D.; Vema, S.; Menkin, S.; Emge, S. P.; Morris, A. J.; Grey, C. P. Forced Disorder in the Solid Solution Li_3P–Li_2S: A New Class of Fully Reduced Solid Electrolytes for Lithium Metal Anodes. *J Am Chem Soc* **2022**, *144* (36), 16350–16365. https://doi.org/10.1021/jacs.2c01913.

(7) Liu, Y.; Peng, H.; Su, H.; Zhong, Y.; Wang, X.; Xia, X.; Gu, C.; Tu, J. Ultrafast Synthesis of I-Rich Lithium Argyrodite Glass–Ceramic Electrolyte with High Ionic Conductivity. *Advanced Materials* **2022**, *34* (3). https://doi.org/10.1002/adma.202107346.

(8) Shim, S.; Park, W. B.; Han, J.; Lee, J.; Lee, B. Do; Lee, J.; Seo, J. Y.; Prabakar, S. J. R.; Han, S. C.; Singh, S. P.; Hwang, C.; Ahn, D.; Han, S.; Park, K.; Sohn, K.; Pyo, M. Optimal Composition of Li Argyrodite with Harmonious Conductivity and Chemical/Electrochemical Stability: Fine-Tuned Via Tandem Particle Swarm Optimization. *Advanced Science* **2022**, *9* (28). https://doi.org/10.1002/advs.202201648.

(9) Adeli, P.; Bazak, J. D.; Park, K. H.; Kochetkov, I.; Huq, A.; Goward, G. R.; Nazar, L. F. Boosting Solid-State Diffusivity and Conductivity in Lithium Superionic Argyrodites by Halide Substitution. *Angewandte Chemie* **2019**, *131* (26), 8773–8778. https://doi.org/10.1002/ange.201814222.

(10) Li, S.; Lin, J.; Schaller, M.; Indris, S.; Zhang, X.; Brezesinski, T.; Nan, C.; Wang, S.; Strauss, F. High-Entropy Lithium Argyrodite Solid Electrolytes Enabling Stable All-Solid-State Batteries. *Angewandte Chemie International Edition* **2023**, *62* (50). https://doi.org/10.1002/anie.202314155.

(11) Rayavarapu, P. R.; Sharma, N.; Peterson, V. K.; Adams, S. Variation in Structure and Li+-Ion Migration in Argyrodite-Type Li6PS5X (X = Cl, Br, I) Solid Electrolytes. *Journal of Solid State Electrochemistry* **2012**, *16* (5), 1807–1813. https://doi.org/10.1007/s10008-011-1572-8.

(12) Zhang, Z.; Zhang, J.; Jia, H.; Peng, L.; An, T.; Xie, J. Enhancing Ionic Conductivity of Solid Electrolyte by Lithium Substitution in Halogenated Li-Argyrodite. *J Power Sources* **2020**, *450*, 227601. https://doi.org/10.1016/j.jpowsour.2019.227601.

(13) Arnold, W.; Buchberger, D. A.; Li, Y.; Sunkara, M.; Druffel, T.; Wang, H. Halide Doping Effect on Solvent-Synthesized Lithium Argyrodites Li6PS5X (X= Cl, Br, I) Superionic Conductors. *J Power Sources* **2020**, *464*, 228158. https://doi.org/10.1016/j.jpowsour.2020.228158.

(14) Subramanian, Y.; Rajagopal, R.; Ryu, K.-S. Synthesis, Air Stability and Electrochemical Investigation of Lithium Superionic Bromine Substituted Argyrodite (Li6-XPS5-XCl1.0Brx) for All-Solid-State Lithium Batteries. *J Power Sources* **2022**, *520*, 230849. https://doi.org/10.1016/j.jpowsour.2021.230849.

(15) Taklu, B. W.; Su, W.-N.; Nikodimos, Y.; Lakshmanan, K.; Temesgen, N. T.; Lin, P.-X.; Jiang, S.-K.; Huang, C.-J.; Wang, D.-Y.; Sheu, H.-S.; Wu, S.-H.; Hwang, B. J. Dual CuCl Doped Argyrodite Superconductor to Boost the Interfacial Compatibility and Air Stability for All Solid-State Lithium Metal Batteries. *Nano Energy* **2021**, *90*, 106542. https://doi.org/10.1016/j.nanoen.2021.106542.

(16) Schlenker, R.; Hansen, A.-L.; Senyshyn, A.; Zinkevich, T.; Knapp, M.; Hupfer, T.; Ehrenberg, H.; Indris, S. Structure and Diffusion Pathways in Li_6PS_5Cl Argyrodite from Neutron Diffraction, Pair-Distribution Function Analysis, and NMR. *Chemistry of Materials* **2020**, *32* (19), 8420–8430. https://doi.org/10.1021/acs.chemmater.0c02418.

(17) Adeli, P.; Bazak, J. D.; Huq, A.; Goward, G. R.; Nazar, L. F. Influence of Aliovalent Cation Substitution and Mechanical Compression on Li-Ion Conductivity and Diffusivity in Argyrodite Solid Electrolytes. *Chemistry of Materials* **2021**, *33* (1), 146–157. https://doi.org/10.1021/acs.chemmater.0c03090.

(18) Patel, S. V.; Banerjee, S.; Liu, H.; Wang, P.; Chien, P.-H.; Feng, X.; Liu, J.; Ong, S. P.; Hu, Y.-Y. Tunable Lithium-Ion Transport in Mixed-Halide Argyrodites Li6–XPS5–XClBrx: An Unusual Compositional Space. *Chemistry of Materials* **2021**, *33* (4), 1435–1443. https://doi.org/10.1021/acs.chemmater.0c04650.

(19) Maughan, A. E.; Ha, Y.; Pekarek, R. T.; Schulze, M. C. Lowering the Activation Barriers for Lithium-Ion Conductivity through Orientational Disorder in the Cyanide Argyrodite Li_6PS_5CN. *Chemistry of Materials* **2021**, *33* (13), 5127–5136. https://doi.org/10.1021/acs.chemmater.1c01170.

(20) Zhou, L.; Zhang, Q.; Nazar, L. F. Li-Rich and Halide-Deficient Argyrodite Fast Ion Conductors. *Chemistry of Materials* **2022**, *34* (21), 9634–9643. https://doi.org/10.1021/acs.chemmater.2c02343.

(21) Hartel, J.; Banik, A.; Gerdes, J. M.; Wankmiller, B.; Helm, B.; Li, C.; Kraft, M. A.; Hansen, M. R.; Zeier, W. G. Understanding Lithium-Ion Transport in Selenophosphate-Based Lithium Argyrodites and Their Limitations in Solid-State Batteries. *Chemistry of Materials* **2023**, *35* (12), 4798–4809. https://doi.org/10.1021/acs.chemmater.3c00658.

(22) Bernges, T.; Culver, S. P.; Minafra, N.; Koerver, R.; Zeier, W. G. Competing Structural Influences in the Li Superionic Conducting Argyrodites $Li_6PS_{5-x}Se_xBr$ ($0 \le x \le 1$) upon Se Substitution. *Inorg Chem* **2018**, *57* (21), 13920–13928. https://doi.org/10.1021/acs.inorgchem.8b02443.

(23) Gautam, A.; Ghidiu, M.; Suard, E.; Kraft, M. A.; Zeier, W. G. On the Lithium Distribution in Halide Superionic Argyrodites by Halide Incorporation in $Li_{7-x}PS_{6-x}Cl_x$. *ACS Appl Energy Mater* **2021**, *4* (7), 7309–7315. https://doi.org/10.1021/acsaem.1c01417.

(24) Zhang, J.; Li, L.; Zheng, C.; Xia, Y.; Gan, Y.; Huang, H.; Liang, C.; He, X.; Tao, X.; Zhang, W. Silicon-Doped Argyrodite Solid Electrolyte Li_6PS_5I with Improved Ionic Conductivity and Interfacial Compatibility for High-Performance All-Solid-State Lithium Batteries. *ACS Appl Mater Interfaces* **2020**, *12* (37), 41538–41545. https://doi.org/10.1021/acsami.0c11683.

(25) Kraft, M. A.; Culver, S. P.; Calderon, M.; Böcher, F.; Krauskopf, T.; Senyshyn, A.; Dietrich, C.; Zevalkink, A.; Janek, J.; Zeier, W. G. Influence of Lattice Polarizability on the Ionic Conductivity in the Lithium Superionic Argyrodites Li_6PS_5X (X = Cl, Br, I). *J Am Chem Soc* **2017**, *139* (31), 10909–10918. https://doi.org/10.1021/jacs.7b06327.

(26) Zhou, L.; Assoud, A.; Zhang, Q.; Wu, X.; Nazar, L. F. New Family of Argyrodite Thioantimonate Lithium Superionic Conductors. *J Am Chem Soc* **2019**, *141* (48), 19002–19013. https://doi.org/10.1021/jacs.9b08357.
